# Extraction Temperatures Shape Water-Soluble Metabolite Profiles of *Nepeta nuda* L. and thus Modulate the Bioactive Properties

**DOI:** 10.3390/metabo16050323

**Published:** 2026-05-13

**Authors:** Desislava Mantovska, Alexandra Kapogianni, Ginka Cholakova, Ivanka Tsacheva, Anton Hinkov, Detelina Petrova, Zlatina Gospodinova, Georgi Antov, Danijela Mišić, Krasimir Rusanov, Mila Rusanova, Kalina Shishkova, Momchil Paunov, Zhenya Yordanova, Miroslava Zhiponova

**Affiliations:** 1 Faculty of Biology, Sofia University “St. Kliment Ohridski”, 1164 Sofia, Bulgaria; d_mantovska@biofac.uni-sofia.bg (D.M.); kapojanni@uni-sofia.bg (A.K.); ginka.cholakova@biofac.uni-sofia.bg (G.C.); itsacheva@biofac.uni-sofia.bg (I.T.); ahinkov@biofac.uni-sofia.bg (A.H.); detelina@biofac.uni-sofia.bg (D.P.); k_shishkova@biofac.uni-sofia.bg (K.S.); m_paunov@uni-sofia.bg (M.P.); jiordanova@biofac.uni-sofia.bg (Z.Y.); 2 Centre of Competence “Sustainable Utilization of Bio-Resources and Waste of Medicinal and Aromatic Plants for Innovative Bioactive Products” (BIORESOURCES BG), 1000 Sofia, Bulgaria; krusanov@abv.bg; 3 Institute of Plant Physiology and Genetics, Bulgarian Academy of Sciences, 1113 Sofia, Bulgaria; zlatina.go@abv.bg (Z.G.); antov8107@abv.bg (G.A.); 4 Department of Plant Physiology, Institute for Biological Research “Siniša Stanković”—National Institute of the Republic of Serbia, University of Belgrade, 11108 Belgrade, Serbia; dmisic@ibiss.bg.ac.rs; 5 Department of Agrobiotechnology, Agrobioinstitute, Agricultural Academy, 1164 Sofia, Bulgaria; milagradeva@abv.bg; 6 Research and Development and Innovation Consortium, 1784 Sofia, Bulgaria

**Keywords:** catmint, extract composition, biological activities, water solvent

## Abstract

**Background:** Plants of the genus *Nepeta* are widely used in ethnomedicine for treating inflammatory disorders due to their rich content of bioactive compounds. This study investigated how extraction temperature specifically affects the bioactive potential of aqueous extracts from wild-grown *Nepeta nuda* L. **Methods:** The previously used maceration approach for this plant was applied at 30–60 °C to flowers, leaves, and stems. Phytochemical profiling included spectrophotometric assays, metabolite identification, and quantification. Biological activities reported for this plant were assessed, including antioxidant, anti-inflammatory, antiviral, antiproliferative, and antibacterial capacities. **Results:** Extraction yield was highest in flowers and leaves, where it increased significantly with rising temperature, while stems were less productive. All plant organs exhibited notable bioactivity falling into two groups: lower temperatures (30 and 40 °C) were optimal for antiviral and anti-inflammatory effects, whereas and higher temperatures (50 and 60 °C) enhanced antioxidant potential. The phytochemical composition, evaluated at representative extraction temperatures, revealed differential accumulation of *p*-coumaric acid and luteolin in all organs at 40 °C, while extraction at 60 °C corresponded to elevated levels of phenolic compounds. Flower extracts were confirmed to have the richest metabolic composition and were therefore subjected to further investigation. Extracts obtained at 40 °C influenced C1q binding, supporting their anti-inflammatory activity, whereas extraction at 60 °C resulted in stronger antiproliferative activity in colon cancer cell line. Antibacterial effects were similar at both temperatures. **Conclusions:** These findings highlight the importance of optimizing extraction conditions for future pharmacological applications of *N. nuda*.

## 1. Introduction

Phytochemicals produced by plants largely reflect the co-evolution between plants and their surrounding environment, which includes competing plant species, pathogens, herbivores, and various abiotic factors [1,2]. The genus *Nepeta* has attracted considerable attention in recent years, mainly due to its nepetalactones, which are known to stimulate behavioral responses in felid species [3]. Nepetalactones are monoterpenoids with dual roles as insect pheromones and repellents against phytophagous insects [4]. It has also been suggested that cats may use these plants to repel mosquito attacks [4].

Notably, feline herpesvirus type 1 (FHV-1) is an alphaherpesvirus that causes severe inflammatory diseases in cats [5]. In this context, Hinkov et al. [6] conducted a detailed analysis of aqueous extracts from flowering plants of *Nepeta nuda* subsp. *nuda* L., demonstrating antiviral activity against herpesviruses. *Nepeta nuda* differs from the extensively studied *N. cataria* (catnip) by the absence or reduced levels of nepetalactones [7]. Nevertheless, nepetalactones and other iridoids have been identified predominantly in *N. nuda* flowers [8,9]. In traditional medicine, *N. nuda* has been used to treat inflammatory conditions of the urinary bladder, prostate gland, mammary gland (udder), and wounds [10]. Extraction has typically been performed through decoction (i.e., boiling in water) or as a tincture using a series of alcohol concentrations [11]. Several studies support water as a green and inexpensive solvent that is highly efficient for extracting antioxidants from *Nepeta* species [8,12,13,14].

Hinkov et al. [6], in their search for molecules associated with antiviral activity, performed NMR (nuclear magnetic resonance) analysis of *N. nuda* aqueous extracts and identified major phenolic acids, including rosmarinic, chlorogenic, gallic, vanillic, caffeic, protocatechuic, ferulic, and cinnamic acids, as well as flavonoids such as cirsimaritin, chrysoeriol, vanillin, rutin, and quercetin. Primary metabolites included sucrose, *α*- and *β*-glucose, and amino acids—alanine, glutamine, leucine, threonine, and valine. Other studies have reported that aqueous extracts from *Nepeta* species are rich in chlorogenic, ferulic, and rosmarinic acids, which correlate with high antioxidant capacity and antiproliferative activity in human colon carcinoma HT29 cells [12]. Aqueous extracts from *N. rtanjensis* Diklić et Milojević exhibited in vitro antioxidant, anti-inflammatory, antihyperglycemic, and antimicrobial activities, associated with high levels of rosmarinic acid and monosaccharides such as glucose and fructose [14]. Rosmarinic acid has also been identified in aqueous extracts of *N. italica* L., where it retained antioxidant and anti-inflammatory effects in an ex vivo model of colon inflammation [13].

Aqueous extracts of *N. nuda* have been reported to exhibit phytotoxic effects, inhibiting the growth of wheat and cucumber [15]. At the same time, these extracts possess antioxidant, antiproliferative, and antiviral activities [6,8,12,16]. Flowers are generally richer in phenolic antioxidants and iridoids compared to leaves [8,9]. The anti-inflammatory activity of aqueous extracts from *N. nuda* flowers and leaves has been demonstrated in vitro by inhibition of the inflammation via the classical pathway of complement activation [9]. Consistently, aqueous flower extracts have shown potent anti-inflammatory effects in vivo (rat hind paw model), following both local and systemic administration [17]. Additionally, aqueous leaf extracts exhibited significant anticancer activity against breast, liver, and colon cancer cell lines, with the strongest cytostatic effect observed in colon cancer cells [16].

Previous reports have indicated that the phytochemical composition of *N. nuda* is significantly influenced by growth conditions, plant organ, and extraction parameters [8,9,18]. We observed that the aqueous extracts exhibiting diverse biological activities were prepared at different extraction temperatures, and the incubation time also varied. For example, phytotoxic (allelopathic) activity was assessed using aerial plant parts subjected to 24 h maceration at room temperature [15]. Antioxidant and antiproliferative activities were evaluated in extracts obtained at higher temperatures, such as 80 °C for 45 min [16] or 60 °C for 24 h [8,16]. Antiviral and anti-inflammatory activities were reported for extracts prepared by maceration at 40 °C for 72 h [6] and for 24 h [9].

Based on existing studies on *N. nuda*, a research gap remains regarding the effects of extraction temperature and incubation time. To standardize aqueous extraction procedures and gain deeper insight into extract composition and associated biological activities, we conducted a systematic screening of *N. nuda* extracts prepared through ultrasound-assisted extraction and maceration with a 24 h incubation period in water, following our earlier studies [8,9,16]. Temperatures ranging from 30 to 60 °C were applied to obtain representative extracts corresponding to low and high extraction temperatures for detailed analysis of their phytochemical composition and in vitro biological activities (antioxidant, anti-inflammatory, antiviral, antiproliferative, and antibacterial). To strengthen our observations, we also compared the effect of temperature across different plant organs (flowers, leaves, and stems).

## 2. Materials and Methods

### 2.1. Plant Material

*Nepeta nuda* subsp. *nuda* L. plants (Bulgaria, Rhodope Mountains, Bekovi Skali, 1320 m a.s.l., coordinates 41.99437188774722, 24.396310265460215) were collected during the flowering period. The voucher specimen (SO108229) has been deposited in the Herbarium of Sofia University “St. Kliment Ohridski,” Sofia, Bulgaria [19].

Plant material from approximately 15–20 plants was air-dried in dark, and flowers, leaves, and stems were collected separately and powdered. Aqueous extracts were prepared using plant material and distilled water in ratio 1 g in 20 mL, followed by treatment in an ultrasound water bath for 20 min and subsequent maceration at 30, 40, 50 and 60 °C for 24 h. After filtration, the water solvent was evaporated through lyophilization at −65 °C (Alpha 1-2 LDplus, Martin Christ Gefriertrocknungsanlagen GmbH, Osterode am Harz, Germany), and the dried extracts were stored at −20 °C to preserve stability. Prior to analyses, the extracts were dissolved in sterile water to obtain a defined stock concentration. For biological activity assays, the extract (100 mg mL^−1^) was diluted in the respective buffer media to concentrations below 10 mg mL^−1^). The only exception was the disk diffusion method for antibacterial testing, where 200 mg mL^−1^ of stock solution was applied directly.

### 2.2. Phytochemical Content of Aqueous Extracts

Quantification of extract yield, as well as total phenolics and flavonoid content in extracts (prepared at 30, 40, 50, and 60 °C) from different plant organs (flowers, leaves, and stems), was performed according to [8]. The yield was expressed as the percentage of extract weight per gram plant dry weight. The quantification of total phenolics [20] and flavonoids [21] was performed using gallic acid and quercetin as standards, respectively.

Extracts prepared at 40 and 60 °C from plant organs (flowers, leaves, and stems) were subjected to gas chromatography–mass spectrometry (GC–MS) analysis for non-polar (fatty acids and derivatives) and polar (organic acids, amino acids, sugars, and phenolic derivatives) metabolites, as described in detail in [9]. An Agilent GC 7890 GC system (Agilent Technologies, Inc., Santa Clara, CA, USA) coupled with an Agilent MD 5975C mass spectral detector (Agilent Technologies, Inc., Santa Clara, CA, USA) were used. Reference standards included nonadecanoic acid (for the non-polar fraction), ribitol (for the polar fraction), and 3,5-dichloro-4-hydroxybenzoic acid (for the polar phenolic fraction). Relative metabolite concentrations in leaf and stem samples (leaf and stem) were expressed as logarithmic values with the flower extract at 40 °C set as the reference (value = 0). Three technical repetitions were performed for each sample, each comprising material pooled from 10 to 15 plants.

Extracts prepared at 40 and 60 °C from plant organs (flowers, leaves, and stems) were also subjected to metabolic profiling using a Dionex UltiMate 3000 ultra-high-performance liquid chromatography (UHPLC) system coupled with diode-array detector (DAD) and a triple quadrupole mass spectrometer with (±)heated electrospray ionization (UHPLC/DAD/(±)HESI–MS2) (Thermo Fisher Scientific, Basel, Switzerland) for quantification of major phenolic acids (caffeic acid, chlorogenic acid, rosmarinic acid, and *p*-coumaric acid), flavonoids (apigenin, luteolin, astragalin/kaempferol-3-O-glucoside, and isoorientin/luteolin-6-C-glycoside), and iridoids (nepetanudoside, 1,5,9-epi-deoxyloganic acid, and nepetalactone), as previously described [22].

### 2.3. Biological Activities of Aqueous Extracts

Extracts prepared at 30, 40, 50 and 60 °C from *N. nuda* organs (flowers, leaves, and stems) were screened using previously described approaches for assessing DPPH radical scavenging activity [23], antiviral activity [8], anti-inflammatory activity [24], and cytotoxicity [8].

The DPPH-radical-scavenging activity was measured using Trolox as a standard, according to the method in [23].

Antiviral activity and cytotoxicity were evaluated as described in detail in [8]. An acyclovir (ACV)-resistant strain of *Simplexvirus humanalpha2* (SvHA2) (family Orthoherpesviridae) (strain DD), obtained from the National Center of Infectious and Parasitic Diseases (NCIPD, Sofia, Bulgaria), was propagated in Madine and Darby bovine kidney (MDBK) cells (obtained from American Type Culture Collection (ATCC, № CCL-22, Manassas, VA, USA)). The maximum nontoxic concentration (MNC) was defined as the highest concentration at which the calculated value for cell viability remained at 100%. The 50% cytotoxic concentration (CC_50_) was determined as the extract concentration required to reduce MDBK cell viability by 50%. The anti-SvHA2 activity of *N. nuda* extracts was assessed by measuring cell viability using a colorimetric MTT assay in two experimental setups: (1) simultaneous addition of the extract with cell inoculation and (2) addition of the extract 1 h post-inoculation. The effective concentration (EC_50_) was determined as the concentration at which 50% of the cells retained their viability. The selectivity index (SI) was calculated as: SI = CC_50_/EC_50_.

The anti-inflammatory potential of the extracts (at a concentration of 1 mg mL^−1^) was evaluated using a hemolytic assay to assess their ability to inhibit the complement system activation (C1q), as described in detail in [24,25].

Extracts prepared at 40 and 60 °C from *N. nuda* flowers were further evaluated to clarify the role of the complement system (C1q) in the anti-inflammatory response. Accordingly, enzyme-linked immunosorbent assay (ELISA) was performed to assess C1q-IgG (IgG, immunoglobulin) interactions, as described in [26], using heat-aggregated human IgG (HAIgG) as a model of immune complexes.

Extracts prepared at 40 and 60 °C from *N. nuda* flowers were also tested for anticancer activity against a murine colon adenocarcinoma cell line, Colon 26 (CLS, Eppelheim, Germany), following the procedure described in [16]. A human non-cancerous skin cell line, BJ (ATCC, USA), was used as a control. Cellular metabolic activity and proliferation were evaluated using an MTT assay 72 h after treatment of the cells with *N. nuda* extracts in a concentration range from 10 to 600 µg mL^−1^. Half-maximal inhibitory concentrations (IC_50_) values for both cancer and non-cancerous cell lines were calculated using GraphPad Prism 8 (GraphPad Software, San Diego, CA, USA). For the clonogenic assay, which evaluates the ability of single cells to proliferate and form colonies or clones after treatment, Colon 26 cells were seeded at a density of 1 × 10^3^ cells per well in 6-well plates. After overnight incubation, the cells were treated with *N. nuda* extracts prepared at 40 and 60 °C for 7 days. Untreated cells cultured in complete medium served as the negative control. At the end of the treatment period, the cells were fixed and stained with 2% methylene blue in 50% ethanol. Colonies consisting of at least 50 cells were counted under a microscope and the colony-forming ability was expressed as a percentage relative to untreated control cells.

Antimicrobial activity was tested as described in detail in [8]. For qualitative screening, the disk diffusion approach was applied. Extracts were loaded onto sterile paper disks (6 mm in diameter) to obtain the final concentration of 10 mg per disk. Inhibition zones were measured after 24 h incubation period at 37 °C, using tetracycline (30 µg/disk) as a positive control. For quantitative assessment, the minimum inhibitory (MIC) and minimum bactericidal concentration (MBC), expressed as mg mL^−1^, were determined. A series of two-fold dilutions (50–0.78 mg mL^−1^, i.e., 50, 25, 12.5, 6.25, 3.125, 1.56, 0.78 mg mL^−1^) was prepared in 96-well microtiter plates. Bacterial growth was assessed spectrophotometrically for MIC determination. As positive controls, penicillin G (30–0.235 µg ml^−1^ for Gram-positive bacteria), and gentamicin at concentrations ranging from 20 to 0.156 µg ml^−1^ for Gram-negative bacteria were used. MBC was determined as the lowest extract concentration inducing 99.9% growth inhibition of the bacterial inoculum after 24 h incubation at 37 °C. To additionally confirm the absence of bacterial growth, 10 µL from the defined MBC were plated on agar MH medium.

The following bacterial strains were purchased from the Bulgarian National Bank for Industrial Microorganisms and Cell Cultures (NBIMCC): the Gram-negative bacteria *Acinetobacter calcoaceticus* NBIMCC 3730 and *Klebsiella pneumoniae* NBIMCC 3670 and the Gram-positive bacteria *Bacillus subtilis* NBIMCC 1709 and *Staphylococcus aureus* ATCC 25923. The inhibition zones were measured after 24 h incubation period at 37 °C.

### 2.4. Statistics

The data represent at least three biological replicates, each comprising a representative sample of approximately 10–20 plants. The results are expressed as mean values ± standard error (SE) with *n* ≥ 3 technical replicates. Statistical comparisons among extraction conditions (30–60 °C) for the studied parameters across all plant organs were performed using two-way analysis of variance (ANOVA), with organ and temperature as factors, followed by Tukey’s post hoc test. Significant differences are indicated by different letters next to the values. Comparisons against control groups were conducted using one-way ANOVA followed by Dunnett’s post hoc test, with significant differences indicated by asterisks. Statistical analyses were carried out using Sigma Plot 11.0 (Systat Software, Inc., Erkrath, Germany), JASP 0.96.0 [27], and GraphPad Prism 8 (GraphPad Software, San Diego, CA, USA). For C1q binding, unpaired *t*-test with Welch’s correction was applied. The results were considered statistically significant at *p* < 0.05.

Principal component analyses (PCA) of the metabolites in and bioactivities of the examined variants were performed using prcomp function from stats package [https://search.r-project.org/R/refmans/stats/html/prcomp.html (accessed on 6 May 2026)] in R 4.5.3 [https://cran.r-project.org/ (accessed on 6 May 2026)] following logarithmic normalization of the experimental values. PCA graphs were plotted using the R package ggbiplot 0.6.2 [https://github.com/friendly/ggbiplot (accessed on 6 May 2026)].

## 3. Results

### 3.1. The Extraction Temperature Affects the Yield and Composition of N. nuda Aqueous Extracts

**Figure 1:**
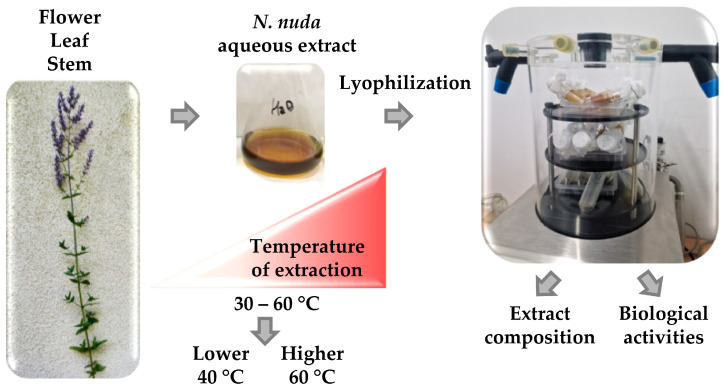
Simplified schematic of the experimental setup for the preparation of *Nepeta nuda* aqueous extracts. A range of extraction temperatures was tested, and two temperatures (40 and 60 °C) were selected for detailed investigation of their effects on metabolite profiles and bioactive properties.

Aqueous extracts from *N. nuda* flowering plants were prepared at different temperatures (30–60 °C) to elucidate how this factor affects the yield of bioactive metabolites and their bioactive potential (Figure 1).

The effect of extraction temperature was monitored in individual plant organs to identify common trends. The extract yield from flowers increased significantly at 60 °C (Table S1). In leaves, the increase occurred at 40 °C, after which the yield remained constant. Stems were the least productive, showing no significant change with increasing temperature.

Total phenolic content appeared highly temperature-sensitive and gradually increased with each tested temperature in flowers and leaves, whereas in stems, a significant accumulation was observed only at 60 °C (Table S1). Compared to total phenolics, flavonoids were the most abundant in leaves and showed a positive correlation with temperature. In flowers, flavonoid content increased at 40 °C and then remained stable at higher temperatures, while in stems, a more pronounced increase was observed at 60 °C.

Based on the screening, we concluded that 30 °C is insufficient for efficient extraction, while 50 °C represents an intermediate condition between 40 and 60 °C in terms of yield and metabolite recovery. Therefore, 40 and 60 °C were selected as representative lower and higher temperatures, respectively, for further detailed investigation (Figure 1). Table 1 highlights the positive correlation between increasing temperature and phenolic content across all plant organs.

For higher resolution, the metabolite composition of the selected extracts prepared at 40 and 60 °C was analyzed using GC–MS (Table S2). A total of 40 polar and six non-polar primary and secondary metabolites were identified. Their relative abundances indicated that flowers and leaves are more enriched in polar metabolites (polyphenolics, amino acids, and sugars), whereas stems contain higher levels of organic acids and non-polar fatty acids. Comparison of extraction temperature dependence across the different organs revealed a trend toward increased metabolite levels upon extraction at 60 °C. Table 2 lists the metabolites showing the most significant changes in the three plant organs.

The extraction of fatty acids did not show strong temperature dependence across the examined plant organs (Table S2). Significant differences were observed in flower extracts for palmitic and stearic acids. At 40 °C, higher levels of cysteine, arabinose, and β-gentiobiose were observed (Table 2).

A more precise quantitative characterization of phenolic acids, flavonoids, and iridoids was achieved using a targeted metabolomics approach (Table 3).

At 60 °C, the content of phenolic acids (chlorogenic, caffeic, and rosmarinic) in flowers and leaves was significantly increased when compared to that at 40 °C. A similar trend was observed for the flavonoid isoorientin (luteolin-6-C-glycoside). In contrast, extraction at 40 °C favored the accumulation of more thermosensitive metabolites, including polyphenolics such as *p*-coumaric acid (in all organs), luteolin (in all organs), and apigenin (in flowers), as well as iridoids such as nepetanudoside and nepetalactone (both in flowers). The content of iridoid 1,5,9-*epi*-deoxyloganic acid was not significantly affected by extraction temperature.

### 3.2. The Extraction Temperature Differentially Modulates the Biological Activities of N. nuda Aqueous Extracts

To check the biological activities of *N. nuda* aqueous extracts, we first evaluated whether their phytochemical composition exerted cytotoxic effects. Cytotoxicity analyses were performed on normal kidney cells. The results did not reveal substantial differences between the variants, although a slight decrease in viability was observed at higher extraction temperatures for leaf and stem extracts (Table 4 and Table S3).

In our previous studies, we reported in vitro biological activities of *N. nuda* [8,9]. A higher extraction temperature (60 °C) significantly enhanced the antioxidant properties of the aqueous extracts, whereas at a lower temperature (40 °C), anti-inflammatory and antiviral activities were optimal and declined sharply with increasing temperature (Table 5 and Table S4).

*Nepeta nuda* extracts exhibited stronger antiviral protection when they were added immediately after inoculating the cell monolayer with SvHA2, i.e., SvHA2^SA^, whereas the activity was weaker when the extracts were added 1 h post-infection, i.e., SvHA2^PA^ (Table 5, Tables S4 and S5). At the same time, one of the most used nucleoside analogs for the treatment of herpes infections, acyclovir (ACV), showed no activity against the same viral model in either experimental setup (Table S6). These results indicate that extracts obtained at temperatures up to 40 °C were the most active against the replication of ACV-resistant SvHA2 strain, likely by interfering with the early stages of the viral replicative cycle.

The selectivity index (SI) reflects the selectivity of an extract toward viral targets relative to cellular toxicity. A plant extract or compound is generally considered to have therapeutic potential when SI ≥ 10 [28]. In the experimental setup involving simultaneous addition of the extracts and the virus, SI values for flower, leaf, and stem extracts obtained at 30 and 40 °C exceeded 10 (Table S5). This criterion was also met by stem extracts obtained at 50 and 60 °C. In contrast, in the post-inoculation setup (addition 1 h after infection), an SI value of 10 was observed only for the stem extract obtained at 30 °C.

**Figure 2:**
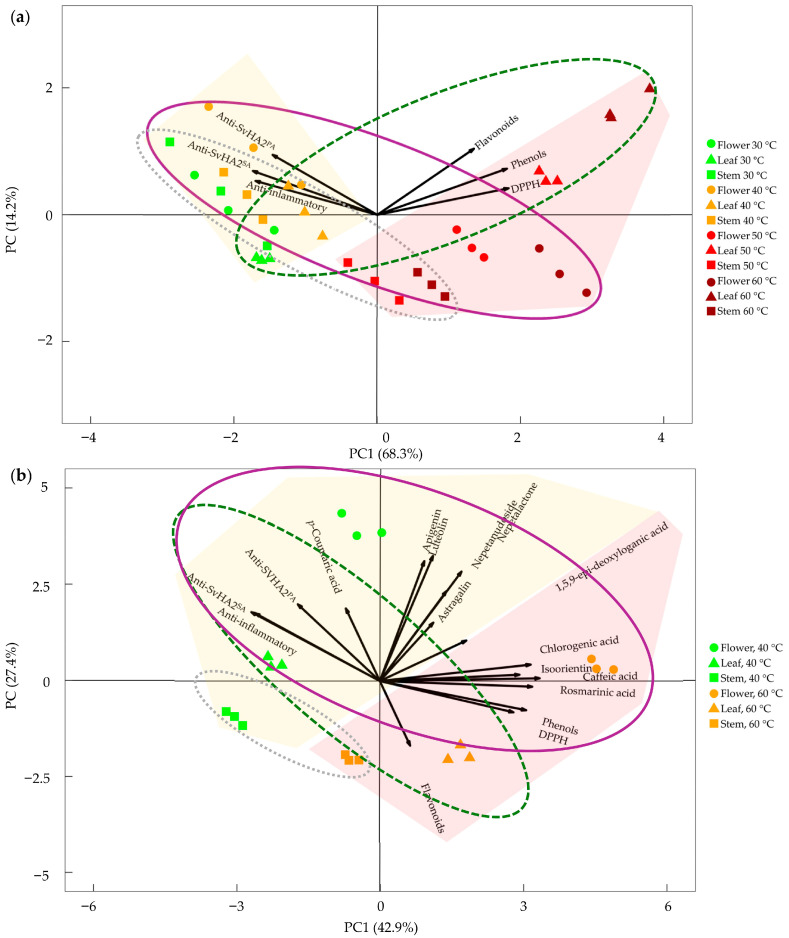
Interdependence between phytochemical composition and biological activities of aqueous extracts from *N. nuda* flower, leaf, stem. (**a**) Principal component analysis (PCA) showing the relationship between total phenolic content and biological activities in extracts prepared across a range of temperatures (30–60 °C). (**b**) PCA illustrating the interdependence between quantified secondary metabolites and biological activities in extracts prepared at 40 and 60 °C. Antiviral activity against *Simplexvirus humanalpha2* (simultaneous application, SvHA2^SA^; 1 h post-treatment, SvHA2^PA^), anti-inflammatory activity (hemolysis and C1q assays), and antioxidant activity (DPPH) are shown. Symbols denote plant organs: circles (flowers), triangles (leaves), and squares (stems). Temperature increase is indicated by a color gradient from green to dark brown. Yellow and light brown shaded areas indicate group distributions associated with lower and higher extraction temperatures, respectively. Ellipses represent the distribution of plant organs: solid purple line (flowers), dashed green line (leaves), and dotted gray line (stems).

The effect of extraction temperature on the relationship between the tested biological activities and the phytochemical composition in the different *N. nuda* organs was summarized in Figure 2.

In the PCA related to biological activities along the extraction temperature gradient (Figure 2a), the first principal component (PC1), accounting for 68.3% of the variance, was associated with all tested parameters. Phenolic content, flavonoids, and DPPH antioxidant capacity increased along PC1, whereas anti-inflammatory and antiviral (SvHA2^SA^ and SvHA2^PA^) activities decreased. The second principal component (PC2) explained an additional 14.2% of the variance, with all tested parameters increasing along this axis.

Elevation of extraction temperature (from 30 to 60 °C) corresponded to systematic shifts in sample positioning along both principal components, supporting the conclusion that temperature influences extraction efficiency and phytochemical composition. Lower extraction temperatures were associated with stronger anti-inflammatory and antiviral properties, whereas higher temperatures were linked to greater antioxidant capacity.

Clear clustering of samples according to plant organ (as indicated by overlapping ellipses in Figure 2) was not observed overall. However, at lower temperatures, organ groups were more similar, while at higher temperatures, they became more distinctly separated, primarily based on flavonoid content: stem extracts showed the lowest levels, leaf extracts the highest, and flower extracts intermediate levels.

Phenolic content, flavonoids, and DPPH activity were positively correlated, as indicated by their similar vector directions. A similar relationship was observed for anti-inflammatory and antiviral (SvHA2^SA^ and SvHA2^PA^) activities. Generally, PCA indicated that temperature is the primary driver of variation in the dataset, while plant organ acts as a secondary factor influencing extract composition, particularly at higher temperatures.

The second PCA, focused on extracts prepared at 40 and 60 °C, further supported temperature-dependent clustering of metabolites and associated biological activities (Figure 2b). This analysis also highlighted the contribution of plant organ, with flowers showing the highest metabolite enrichment, followed by leaves and stems.

### 3.3. Anti-Inflammatory C1q Inhibitory Potential of Aqueous Extracts from N. nuda Flowers

**Figure 3:**
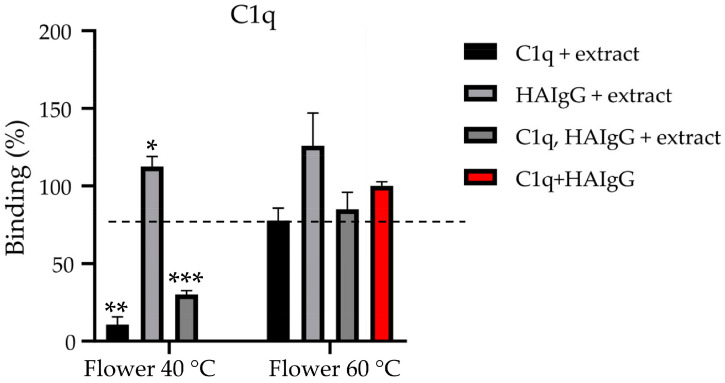
Effect of aqueous extracts of *N. nuda* flowers (prepared at 40 and 60 °C) on the interaction between C1q and immunoglobulin G (HAIgG). Data are presented as mean ± SD (*n* = 3). Statistical differences were assessed using an unpaired *t*-test with Welch’s correction and are indicated by asterisks: * (*p* ≤ 0.05), ** (*p* ≤ 0.01), and *** (*p* ≤ 0.001). The positive control (red) represents the interaction between untreated C1q and HAIgG, defined as 100% binding.

Flowers are generally enriched in metabolites and several reports demonstrated their bioactive potential. In line with this, our previous work on *N. nuda* aqueous extracts highlighted their anti-inflammatory activity mediated via the complement system (Table 5; [11]). Based on this evidence, and particularly on the inhibitory effect of *N. nuda* aqueous extracts on the complement system as demonstrated by the hemolysis assay (Table 5), we aimed to further elucidate their effect on the classical complement pathway. Activation of the classical pathway is initiated by the interaction of serum C1q with immune complexes, represented by heat-aggregated human IgG (HAIgG). The role of extraction temperature was investigated using an ELISA by treating C1q and HAIgG individually with *N. nuda* extracts prepared at 40 and 60 °C, as well as by treating both components prior to their interaction (Figure 3).

Binding between extract-treated C1q with HAIgG was completely inhibited when the 40 °C extract was used, suggesting a significant conformational change in C1q induced by the plant extract.

In contrast, the interaction between C1q and extract-treated HAIgG was comparable to the positive control. Nevertheless, physiologically relevant scenario involves simultaneous exposure of both C1q and IgG to the extract, which resulted in suppression of inflammatory signaling. Under these conditions, when both proteins were treated with the extract, the formation of C1q–HAIgG complexes decreased approximately fourfold. In contrast, extracts prepared at 60 °C did not exhibit an inhibitory effect on C1q activity.

### 3.4. Anti-Colon Cancer Potential of Aqueous Extracts from N. nuda Flowers

**Figure 4:**
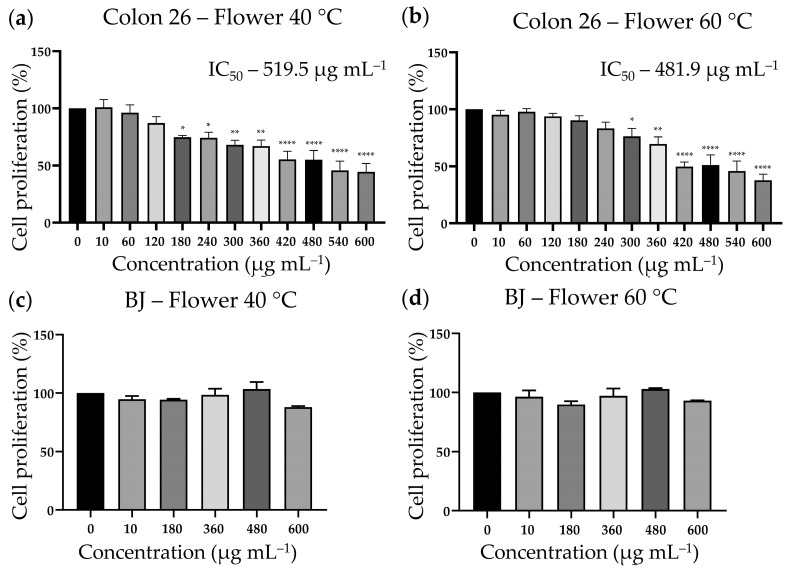
Antiproliferative activity of aqueous extracts of *N. nuda* flowers (prepared at 40 and 60 °C). Statistical differences between untreated controls and treated groups were evaluated by one-way ANOVA followed by Dunnett’s post hoc test and are indicated by asterisks (* *p* ≤ 0.05, ** *p* ≤ 0.01, and **** *p* ≤ 0.0001). (**a**,**b**) Colon 26 colon adenocarcinoma cell line; (**c**,**d**) non-cancerous dermal cell line BJ.

Recently, aqueous extracts from *N. nuda* leaves were investigated to elucidate the mechanism underlying their antitumour effects on the colon adenocarcinoma cell line, Colon 26 [17]. In the present study, we compared the cytostatic properties of aqueous flower extracts of *N. nuda* (prepared at 40 and 60 °C) in the Colon 26 cell line and the non-cancerous dermal cell line BJ. The results demonstrated a statistically significant antiproliferative effect of both extracts on Colon 26 cancer cells (Figure 4a,b).

Lower concentrations of the flower extract prepared at 40 °C exerted antiproliferative effect; however, the extract prepared at 60 °C demonstrated a stronger cell growth inhibitory potential—IC_50_ 481.9 µg mL^−1^ at 60 °C, and IC_50_ 519.5 µg mL^−1^ at 40 °C. The greatest reduction in cell proliferation was reached at the highest applied concentration (600 µg mL^−1^), reaching 37.81% and 44.43% of control cell growth levels for extracts prepared at 60 °C and 40 °C, respectively.

Both *N. nuda* extracts did not show any substantial inhibitory effect on the proliferation of the non-cancerous BJ cell line, indicating high selectivity of their cytostatic anticancer action (Figure 4c,d). Lowest and highest extract concentrations were tested, and at the highest tested concentration (600 µg mL^−1^), cell proliferation remained at 87.91% for the extract prepared at 40 °C and 93.18% for the extract prepared at 60 °C.

**Figure 5:**
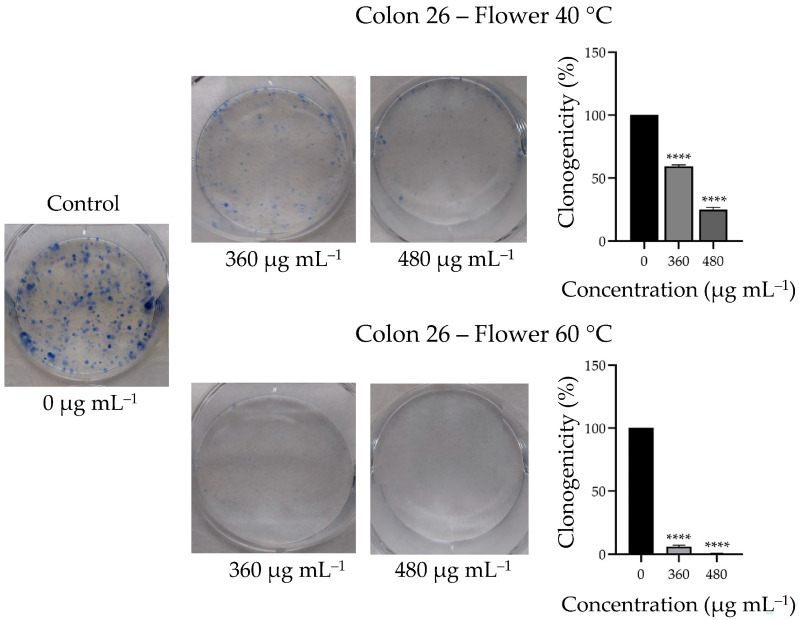
Effects of aqueous extracts of *N. nuda* flowers (prepared at 40 and 60 °C) on the clonogenicity of Colon 26 cells. Cells were treated for 7 days with 360 and 480 µg mL^−1^ and compared to untreated control. Graphs represent quantitative evaluation of Colon 26 cells clonogenicity. Data are presented as mean ± error bars. Asterisks indicate significant differences compared to the control group, determined by one-way ANOVA followed by Dunnett’s post hoc test (**** *p* < 0.0001).

As a next step, the long-term proliferative capacity of colon cancer cells was evaluated following 7-day treatment with flower extracts prepared at 40 and 60 °C at concentrations of 360 and 480 µg mL^−1^, which are close to the IC_50_ values determined by the MTT assay. A dose-dependent anticlonogenic effect of the extracts was observed, which was markedly stronger for the extract prepared at 60 °C compared to that at 40 °C (Figure 5).

At a concentration of 360 µg mL^−1^, clonogenicity of tumor cells was reduced to 59.32% by the extract prepared at 40 °C, whereas the extract prepared at 60 °C resulted in near-complete inhibition, reducing clonogenicity to 5.93%. At 480 µg mL^−1^, the colony-forming ability of cancer cells was further reduced to 25.05% and 0.75% for the extracts prepared at 40 °C and 60 °C, respectively.

### 3.5. Antibacterial Activity of Aqueous Extracts from N. nuda Flowers

Since methanolic extracts of *N. nuda* have previously been reported to exhibit antibacterial activity against both Gram-negative and Gram-positive bacteria [8], we also evaluated the antibacterial potential of aqueous extracts. Overall, aqueous extracts of *N. nuda* flowers (prepared at 40 and 60 °C) showed bactericidal activity against three of the tested bacterial strains—*A. calcoaceticus*, *S. aureus*, and *B. subtilis*—while no effect was observed against *K. pneumoniae*. The results from the qualitative disk diffusion assay demonstrated the strongest inhibitory effects against *B. subtilis* and *A. calcoaceticus* at both extraction temperatures (Table 6).

The activity of the extracts against *S. aureus* was more variable. Quantitative analysis revealed MIC values ranging from 6.25 to 25 mg mL^−1^ and MBC values from 6.25 to 50 mg mL^−1^. The Gram-positive bacteria *B. subtilis* and *S. aureus* exhibited the highest sensitivity, followed by the Gram-negative *A. calcoaceticus*.

Extraction temperature did not significantly affect antibacterial activity overall, although some trends were observed. *Bacillus subtilis* showed increased sensitivity to the extract prepared at 40 °C (MIC/MBC = 6.25/6.25 mg mL^−1^), whereas *S. aureus* was more sensitive to the extract prepared at 60 °C (MIC/MBC = 6.25/12.5 mg mL^−1^). In the case of *A. calcoaceticus*, a significant difference was observed only in the disk diffusion assay, with stronger inhibition exhibited by the extract prepared at 40 °C.

### 3.6. Metabolite–Bioactivity Correlation

To identify metabolites associated with the temperature-dependent biological activities, a correlation analysis was performed. Only statistically significant correlations are presented in Table 7.

**Figure 6:**
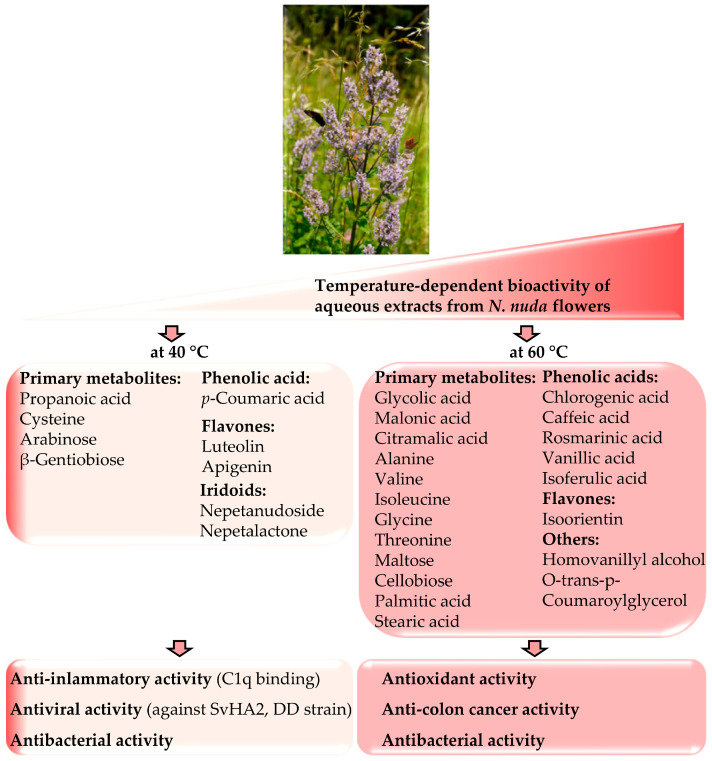
Model for temperature-dependent bioactivity of aqueous extracts of *N. nuda* flowers.

The compositions and biological activities of the extracts prepared at 40 and 60 °C are summarized in Figure 6.

## 4. Discussion

This study investigated how extraction temperature influences the bioactive properties of aqueous extracts of *N. nuda*. The analyses of flowers, leaves, and stems confirmed bioactivity in all parts, with flowers and leaves exhibiting higher phytochemical content; notably, flowers contained the highest levels of iridoids. Extraction at 40 °C optimized antiviral and anti-inflammatory activities, which were associated with elevated levels of *p*-coumaric acid, luteolin, apigenin, nepetanudoside, nepetalactone (Figure 6). In contrast, extraction at 60 °C enhanced antioxidant activity, correlating with increased concentrations of caffeic and rosmarinic acids, as well as primary metabolites. Flower extracts obtained at 40 °C affected C1q binding, while those extracted at 60 °C demonstrated stronger antiproliferative effects. Antibacterial activity remained comparable at both temperatures. Overall, extraction temperature shapes the metabolic profile and bioactivity of *N. nuda*, underscoring the importance of optimizing extraction conditions for pharmacological applications.

### 4.1. Aqueous Extraction Parameters

A growing emphasis in extraction techniques is on the reduction in hazardous substances, particularly toxic solvents, in order to protect human health and the environment while minimizing energy consumption [29]. Water represents an environmentally friendly solvent, and several studies on *Nepeta* species have employed maceration as a conventional method [8,12]. A highly efficient alternative—subcritical water extraction (SWE), which involves short exposure to elevated temperature and pressure—has recently gained attention [14,30]. A review of water-based extraction applied to *Nepeta* species reveals considerable variation in both temperature and duration. Lower-temperature approaches refer to range from room temperature to 40 °C, with or without repeated 24 h extraction cycles [6,8,9,15]. Higher-temperature methods include maceration or boiling at 60 °C and 80 °C (short treatments of approximately 15 min, with or without repetition, or extended up to 24 h) [8,12,13], as well as SWE at 130 °C for 30 min under pressure [14].

Higher temperatures can weaken cell wall integrity, facilitating the release of insoluble polyphenols and flavonoids from the plant matrix [29]. Prolonged extraction time may further enhance this effect by promoting cell wall loosening. Based on previous reports and our earlier work on *N. nuda*, the present study employed a 24 h maceration approach, which has been shown to be sufficient and effective for extracting bioactive compounds [8,9].

In *N. nuda*, water has been reported to yield a higher amount of dry extract compared to methanol, ethanol, acetone, or chloroform [8], and our results further demonstrate that extraction yield increases with temperature. At temperatures below 40 °C, the yield was too low, whereas above 60 °C no further increase was observed, which guided the selection of this temperature range. The physicochemical properties of water—such as dielectric constant, surface tension, and viscosity—change with temperature, leading to decreased polarity [31]. Consequently, extraction at higher temperatures becomes less selective and can solubilize moderately polar and even non-polar compounds [32], which may explain higher extract yield observed at 60 °C. The differences in biological activity between lower and higher temperatures suggest thermo-sensitivity of certain compounds or molecular complexes, resulting in selective extraction effects that warrant further investigation.

### 4.2. Differential Phytochemical Content of Aqueous Extracts

Increasing water temperature enhances vapor pressures, thermal desorption, and diffusion rates of compounds. Elevated temperatures improve the solubility and diffusion of phenolic compounds, thereby accelerating extraction, although they may also lead to compound degradation [29]. Both our results and previous studies indicate that rosmarinic and caffeic acids are more abundant in extracts obtained at moderate to high temperatures [8,12,13,14]. Subcritical water extraction (SWE) has been reported to increase rosmarinic acid yield by up to 41% at elevated temperatures [30]. Notably, rosmarinic and other phenolic acids have also been detected at 40 °C [6], likely due to prolonged extraction (three 24 h cycles), use of whole aboveground plant material, and differences in plant origin.

Regardless of the plant organ, extraction of most phenolic acids (caffeic, chlorogenic, and rosmarinic acids) within this study was more efficient at 60 °C. An exception was *p*-coumaric acid, the least polar phenolic acid examined, which was more efficiently extracted at 40 °C. This finding was surprising, considering that all targeted phenolic acids belong to the hydroxycinnamic acid group, share structurally similarities, and exhibit comparable physicochemical properties. Furthermore, *p*-coumaric acid is a key precursor in the biosynthesis of caffeic acid and its derivatives, namely ferulic acid, coniferyl alcohol, and sinapyl alcohol, all of which represent significant building blocks in lignin [33]. It has been suggested that increased phenolic acids content at higher temperatures may result from the cleavage of lignin–phenolic acid bonds or partial lignin degradation [34]. However, this does not appear to apply in the present study, as *p*-coumaric acid content decreased at 60 °C.

At 60 °C, reduced levels of *p*-coumaric acid, flavone aglycones (luteolin and apigenin), and monoterpenoids (nepetanudoside and nepetalactone) were observed. Luteolin and *p*-coumaric acid showed notable variability across all plant organs, whereas other metabolites were more specific to flowers. Thermal degradation is a common explanation for reduced polyphenol content at elevated temperatures [31]. However, the response to temperature appears to be compound-specific and may reflect differences in solubility (polarity) and thermal properties, such as diffusivity and vapor pressure.

Flavones are generally most abundant in *N. nuda* flowers [8,9], which was confirmed in this study. Flavone aglycones (luteolin and apigenin) were more efficiently extracted at 40 °C. In contrast, flavonoid glycosides—kaempferol-3-O-glucoside (astragalin) and luteolin-6-C-glycoside (isoorientin)—were extracted with similar efficiency at both 40 °C and 60 °C, with minor exceptions. These findings suggest greater thermal stability of glycosylated flavonoids compared to their aglycone counterparts.

Iridoids, characterized by a partially hydrogenated cyclopenta-[c]pyran system, are efficiently extracted using polar solvents such as water [35]. Hot water (50–100 °C) has been shown to be effective for extracting iridoid glycosides, including aucubin and catalpol [36]. This was also supported by the present study, although no significant difference in the extraction efficiency of the predominant *N. nuda* iridoid, 1,5,9-epi-deoxyloganic acid, was observed between 40 °C and 60 °C. The iridoid glycoside nepetanudoside exhibited slight instability at 60 °C, but only in flowers. The iridoid aglycone nepetalactone is a volatile compound with a boiling point of 71 °C at 0.05 mmHg, making it prone to evaporation even at relatively low temperatures [4]. Therefore, its lower content in samples extracted at 60 °C may be attributed to increased volatilization.

Higher temperatures also promote the extraction of other polar metabolites, including organic acids, amino acids, and sugars. Overall, extract bioactivity likely reflects synergistic or antagonistic interactions among multiple constituents [6,29].

### 4.3. Differential Biological Activities in Aqueous Extracts

The viral model used in this study is a laboratory-adapted isolate optimized for replication in the employed cell line. The observed antiviral activity is consistent with previous findings on total aqueous extracts of *N. nuda* (extracted at 40 °C) [6], where experiments targeting different stages of viral replication indicated that the effect is associated with the viral adsorption stage. Based on these findings, it can be assumed that extracts from flowers, leaves, and stems primarily inhibit viral adsorption. Flavones exhibit diverse biological activities in animals, partly determined by their lipophilicity and ability to cross cellular membranes, which depend on the number and position of hydroxyl groups. Luteolin and apigenin, containing four and three hydroxyl groups, respectively, are relatively hydrophilic. In the present study, the correlation between antiviral activity and flavone content supports their role in inhibiting viral adsorption to cell membranes, potentially acting synergistically with other compounds such as cysteine, nepetanudoside, nepetalactone, and *p*-coumaric acid [37,38,39]. Importantly, the observed activity against an acyclovir-resistant strain suggests that these extracts are not only effective against herpesviruses but also against strains resistant to one of the primary antiviral drugs used in therapy.

Flavones are known to interact with DNA, RNA, and proteins, contributing to a wide range of biological effects [40]. Apigenin, for instance, binds multiple cellular targets, including those in cancer cells. Although flavones are widely studied for their anti-inflammatory, anticancer, and antimicrobial properties, their mechanisms of action remain incompletely understood. In this study, *N. nuda* flower extracts influenced C1q in the classical complement pathway, possibly through flavones or flavone–protein complexes. Notably, extracts obtained at 40 °C exhibited a pronounced anti-inflammatory effect, in contrast to those obtained at 60 °C. These differences correspond to variations in metabolite composition, with luteolin and *p*-coumaric acid present at higher levels in the 40 °C extracts across all plant organs. Although neither compound is known to directly interact with C1q or IgG, this study provides, to the best of our knowledge, the first indication that *N. nuda* extracts may exert anti-inflammatory effects by modulating the initial steps of the classical complement activation pathway.

Flavones and *p*-coumaric acid also exhibit antioxidant activity [39,40]. Luteolin inhibits xanthine oxidase, while apigenin reduces reactive oxygen species and protects against inflammation-induced damage [40]. Many of their anti-inflammatory and antimicrobial effects are associated with modulation of the Toll-like receptor (TLR)/NF-κB signaling pathway, which regulates key mediators such as tumor necrosis factor (TNF), interleukin-1 (IL-1), and cyclooxygenase-2 (COX-2). The similarities between plant and mammalian defense pathways suggest the presence of evolutionarily conserved molecular targets. Because NF-κB and COX-2 are often upregulated in cancer and inflammatory conditions, flavones may exert their effects through shared mechanisms, including the induction of apoptosis. Moreover, *p*-coumaric acid seems to act synergistically as it decreases the expression of the inflammatory mediators such as TNF and IL-6 [41].

Glycosides of luteolin and apigenin were previously identified in extracts prepared at higher temperatures [42]. Consistently, luteolin-6-C-glucoside (isoorientin) was more abundant in *N. nuda* aqueous extracts obtained at 60 °C. In general, flavonoid glycosides exhibit weaker anti-inflammatory activity than their aglycone counterparts, likely due to reduced cellular uptake [42].

In the present study, flower extracts obtained at 40 °C showed weaker antiproliferative activity, which may be related to their flavone profile. Aglycones such as luteolin and apigenin are generally more bioavailable than their glycosylated forms, as they are more readily absorbed in the intestine [43]. In contrast, rosmarinic, chlorogenic, and ferulic acids have been identified as key antiproliferative compounds in *N. nuda* aqueous extracts [12]. Previous in vitro studies on human cancer cell lines (including breast, colon, and hepatocellular carcinoma) have shown that *N. nuda* leaf extracts inhibit proliferation and induce apoptosis [16]. In agreement with these findings, the present study demonstrates the antiproliferative potential of flower extracts.

Regarding antibacterial activity, aqueous extracts of *Nepeta cataria* leaves and stems prepared at room temperature were previously reported to lack activity against *S. aureus* and *B. subtilis* [44]. In contrast, in the present study, *N. nuda* flower extracts obtained at 40 °C exhibited moderate bactericidal activity, particularly against Gram-positive bacteria. This difference may be explained by the presence of an additional outer membrane rich in lipopolysaccharides in Gram-negative bacteria, which limits compound penetration [45]. Interestingly, significant growth inhibition of the Gram-negative bacterium *A. calcoaceticus* was observed, which was not reported before for aqueous extracts. Increasing the extraction temperature to 60 °C had little effect on antibacterial activity. Methanol and chloroform extracts of *N. nuda* prepared at 60 °C have demonstrated broader antibacterial activity against *A. calcoaceticus*, *K. pneumoniae*, *B. cereus*, and *S. aureus* [10,20]. These findings suggest that aqueous extracts have a narrower range of bioactive compounds, and that extraction temperature has a limited impact on their antibacterial efficacy.

## 5. Conclusions

Standardization of extract preparation is essential not only for reproducibility but also for ensuring consistent extract composition and associated biological activities. In this study, extraction temperature was identified as a major factor influencing the bioactivity of *N. nuda* aqueous extracts. At lower temperatures, specific metabolites (e.g., luteolin, *p*-coumaric acid) were preferentially extracted, contributing to enhanced antiviral and anti-inflammatory potential. In contrast, higher temperatures favored the extraction of phenolic acids and a broader range of primary metabolites, resulting in stronger antioxidant and antiproliferative activity (Figure 6). Extraction temperature had a limited impact on antibacterial efficacy. Furthermore, a 24 h maceration approach proved to be efficient for the extraction of bioactive compounds. Future studies exploring additional parameters—such as alternative extraction techniques (e.g., subcritical water extraction and ultrasound-assisted extraction) and shorter extraction times—may provide further optimization for practical applications.

The key contribution of this work lies in establishing a clear relationship between the identified and quantified metabolites and the observed biological activities. These findings provide a solid foundation for future research aimed at identifying specific bioactive compounds and elucidating their mechanisms of action.

## Figures and Tables

**Table 1:** Temperature-dependent yield and total phenolic content in aqueous extracts from *N. nuda* flowers, leaves, and stems. Data are presented as mean values and SE (*n* ≥ 3). Statistical differences between variants were determined by two-way ANOVA followed by Tukey’s post hoc test and are indicated with different letters. For visualization, values are represented on a color scale ranging from white (minimum) to red (maximum).

Extraction t °C	40 °C	60 °C
**Yield %**		
Flower	16.6 ± 1.8 ^b^	20.7 ± 0.7 ^c^
Leaf	19.7 ± 0.4 ^c^	21.4 ± 0.6 ^c^
Stem	11.9 ± 0.5 ^a^	12.9 ± 0.5 ^a^
**Phenols mg g DW^−1^**		
Flower	70.24 ± 0.65 ^b^	91.07 ± 0.56 ^c^
Leaf	63.67 ± 1.40 ^a^	104.69 ± 0.88 ^d^
Stem	60.06 ± 1.21 ^a^	68.30 ± 0.81 ^b^
**Flavonoids mg g DW^−1^**		
Flower	40.26 ± 0.40 ^a^	43.51 ± 0.35 ^ab^
Leaf	55.26 ± 0.26 ^c^	84.82 ± 1.52 ^d^
Stem	43.16 ± 0.70 ^ab^	46.67 ± 0.53 ^b^

**Table 2:** Metabolites identified by GC–MS in aqueous extracts from *N. nuda* flowers, leaves, and stems. Relative values are expressed as logarithmic ratios normalized to a control sample (flower extract at 40 °C). Statistical differences between variants for each metabolite were determined by two-way ANOVA followed by Tukey’s post hoc test and are indicated by different letters. For visualization, values for each metabolite are presented on a color scale ranging from white (minimum) to red (maximum). Numbers correspond to metabolites listed in Table S2 (P, polar).

		RT	RI	Flower		Leaf		Stem	
				40 °C	60 °C	40 °C	60 °C	40 °C	60 °C
	**Organic acids**								
P2	Glycolic acid	5.1120	1073.6	0 ^b^	0.38 ^d^	−0.27 ^a^	0.26 ^cd^	0.11 ^bc^	1.04 ^e^
P3	Malonic acid	6.511	1198.2	0 ^d^	0.72 ^e^	−0.34 ^c^	−0.01 ^d^	−1.33 ^a^	−0.62 ^b^
P6	Fumaric acid	7.952	1346.3	0 ^ab^	0.21 ^b^	−0.19 ^a^	0.32 ^b^	0.70 ^c^	1.31 ^d^
P8	Citramalic acid	9.050	1456.5	0 ^a^	0.73 ^c^	0.29 ^b^	0.35 ^b^	0.21 ^ab^	0.62 ^c^
	**Amino acids**								
P13	Alanine	5.442	1101.9	0 ^ab^	1.27 ^d^	0.33 ^b^	1.04 ^cd^	−0.43 ^a^	0.40 ^bc^
P15	Valine	6.639	1211.1	0 ^b^	0.88 ^c^	0.14 ^b^	0.60 ^c^	−0.76 ^a^	−0.02 ^b^
P16	Isoleucine	7.410	1290.4	0 ^b^	0.80 ^d^	0.09 ^bc^	0.49 ^cd^	−0.69 ^a^	−0.20 ^b^
P24	Cysteine	11.762	1694.5	0 ^b^	−1.26 ^a^	1.22 ^e^	0.22 ^bc^	0.47 ^d^	0.42 ^cd^
	**Sugar derivatives**								
P26	Arabinose	11.107	1645.0	0 ^c^	−0.53 ^a^	−0.10 ^c^	−0.50 ^ab^	−0.22 ^bc^	−0.58 ^a^
P34	β-gentiobiose	28.882	2818.8	0 ^bc^	−0.58 ^a^	0.45 ^d^	0.16 ^cd^	−0.07 ^bc^	−0.22 ^b^
	**Phenolic derivatives**								
P36	Vanillic acid	12.564	1 755.0	0 ^ab^	1.31 ^c^	0.28 ^b^	0.41 ^b^	−0.64 ^a^	0.68 ^bc^
P37	Isoferulic acid	17.675	2 086.4	0 ^c^	0.52 ^d^	0.19 ^cd^	0.50 ^d^	−1.05 ^a^	−0.45 ^b^
P38	Caffeic acid	18.419	2132.0	0 ^c^	2.42 ^e^	−1.44 ^b^	1.36 ^d^	−3.19 ^a^	0.04 ^c^
P39	O-trans-p-Coumaroylglycerol	24.033	2486.1	0 ^c^	0.92 ^d^	0.15 ^c^	1.65 ^e^	−2.42 ^a^	−1.05 ^b^

**Table 3:** UHPLC/DAD/(±)HESI–MS^2^ quantification of secondary metabolites in aqueous extracts from *N. nuda* flowers, leaves, and stems. Data are presented as mean ± SE (*n* ≥ 3) and expressed as µg per 100 mg of dry weight (DW). Statistical differences between variants for each metabolite were determined by two-way ANOVA followed by Tukey’s post hoc test and are indicated by different letters. For visualization, quantitative data are shown on a color scale ranging from white (minimum) to red (maximum), separately for phenolic acids (a), flavonoids (b), and iridoids (c).

**(a)** Phenolic Acids										
**Phenolic Acids**	**Chlorogenic Acid**			**Caffeic Acid**		***p*** **-Coumaric Acid**			**Rosmarinic Acid**	
**Extraction t (°C)**	**40 °C**	**60 °C**		**40 °C**	**60 °C**	**40 °C**		**60 °C**	**40 °C**	**60 °C**
Flower	0.81 ± 0.30 ^bc^	1.91 ± 0.46 ^d^		13.11 ± 0.26 ^b^	50.08 ± 6.46 ^d^	6.66 ± 0.04 ^c^		5.15 ± 0.30 ^b^	11.56 ± 2.33 ^a^	206.49 ± 17.36 ^c^
Leaf	0.13 ± 0.05 ^a^	0.87 ± 0.22 ^c^		2.04 ± 0.07 ^a^	27.08 ± 1.16 ^c^	9.18 ± 0.20 ^d^		5.15 ± 0.40 ^b^	2.32 ± 0.25 ^a^	77.40 ± 5.36 ^b^
Stem	0.17 ± 0.04 ^ab^	0.65 ± 0.02 ^abc^		1.07 ± 0.02 ^a^	8.12 ± 0.25 ^ab^	4.55 ± 0.29 ^b^		2.70 ± 0.24 ^a^	5.00 ± 0.05 ^a^	8.63 ± 0.09 ^a^
**(b)** Flavonoids										
**Flavonoids**	**Astragalin**			**Isoorientin**		**Luteolin**			**Apigenin**	
**Extraction t (°C)**	**40 °C**	**60 °C**		**40 °C**	**60 °C**	**40 °C**		**60 °C**	**40 °C**	**60 °C**
Flower	57.55 ± 10.83 ^b^	46.95 ± 5.00 ^b^		0.03 ± 0.01 ^a^	2.30 ± 0.12 ^c^	4.30 ± 0.03 ^f^		2.68 ± 0.14 ^e^	5.90 ± 1.71 ^c^	2.96 ± 0.17 ^b^
Leaf	66.51 ± 13.70 ^b^	58.64 ± 12.88 ^b^		0.02 ± 0.01 ^a^	0.24 ± 0.01 ^b^	1.51 ± 0.11 ^d^		0.81 ± 0.12 ^c^	0.73 ± 0.39 ^a^	0.17 ± 0.15 ^a^
Stem	6.28 ± 2.60 ^a^	19.32 ± 2.52 ^a^		0.02 ± 0.01 ^a^	0.03 ± 0.03 ^a^	0.42 ± 0.05 ^b^		0.12 ± 0.03 ^a^	0.32 ± 0.24 ^a^	0.03 ± 0.01 ^a^
**(c)** Iridoids										
**Iridoids**	**Nepetanudoside**				**1,5,9-epi-deoxyloganic acid**				**Nepetalactone**	
**Extraction t (°C)**	**40 °C**		**60 °C**		**40 °C**		**60 °C**		**40 °C**	**60 °C**
Flower	13,861.71 ± 2044.25 ^c^		10,232.65 ± 298.28 ^b^		300.02 ± 93.46 ^b^		342.45 ± 34.85 ^b^		27.56 ± 0.26 ^c^	21.75 ± 0.22 ^b^
Leaf	10,118.51 ± 144.96 ^b^		10,751.44 ± 646.29 ^b^		113.81 ± 1.09 ^a^		116.11 ± 28.58 ^a^		0.54 ± 0.10 ^a^	0.82 ± 0.04 ^a^
Stem	4053.20 ± 926.39 ^a^		5930.50 ± 1317.16 ^a^		107.52 ± 2.97 ^a^		323.15 ± 0.80 ^b^		0.63 ± 0.03 ^a^	0.71 ± 0.10 ^a^

**Table 4:** Cytotoxicity of aqueous extracts from *N. nuda* flowers, leaves, and stems. Madin–Darby bovine kidney (MDBK) cells were exposed to a range of extract concentrations. Data are presented as mean ± SE (*n* ≥ 3). Statistical differences between variants were determined by two-way ANOVA followed by Tukey’s post hoc test and are indicated by different letters. For visualization, values are shown on a color scale ranging from white (minimum) to red (maximum). CC_50_ denotes the extract concentration required to reduce cell viability by 50%.

Extraction t (°C)	40 °C	60 °C
**Cytotoxicity (mg mL^−1^), CC_50_**		
Flower	5.57 ± 0.57 ^a^	6.24 ± 0.55 ^abc^
Leaf	6.85 ± 0.74 ^abc^	7.64 ± 0.25 ^c^
Stem	6.22 ± 0.48 ^ab^	7.40 ± 0.49 ^bc^

**Table 5:** Biological activities of aqueous extracts from *N. nuda* flowers, leaves, and stems. Data about antioxidant activity (DPPH), anti-inflammatory activity (hemolysis and C1q assays), antiviral activity against *Simplexvirus humanalpha2* (simultaneous application, SvHA2^SA^; 1 h post-treatment, SvHA2^PA^) are presented as mean ± SE (*n* ≥ 3). Statistical differences between variants were determined by two-way ANOVA followed by Tukey’s post hoc test and are indicated by different letters. For visualization, values for each biological activity are displayed on a color scale ranging from white (minimum) to red (maximum).

Extraction t (°C)	40 °C	60 °C
**DPPH mM g DW^−1^**		
Flower	652.51± 5.70 ^b^	1172.45± 4.27 ^c^
Leaf	391.82± 0.71 ^a^	1269.79 ± 92.97 ^c^
Stem	437.41± 2.14 ^a^	668.18 ± 12.82 ^b^
**Anti-Inflammatory Activity %**		
Flower	90.72 ± 4.40 ^b^	18.30 ± 10.60 ^a^
Leaf	98.83 ± 5.40 ^b^	26.30 ± 11.60 ^a^
Stem	90.29 ± 6.40 ^b^	28.54 ± 12.60 ^a^
**Antiviral Protection % Simultaneous Application/SvHA2^SA^**		
Flower	89.93 ± 12.9 ^b^	65.68 ± 3.98 ^a^
Leaf	84.59 ± 0.33 ^b^	68.64 ± 2.79 ^a^
Stem	88.38 ± 2.58 ^b^	72.50 ± 0.70 ^a^
**Antiviral Protection % Post-Infection Application/SvHA2^PA^**		
Flower	68.29 ± 6.51 ^c^	28.22 ± 2.33 ^a^
Leaf	37.18 ± 7.76 ^ab^	41.25 ± 1.06 ^b^
Stem	56.61 ± 4.86 ^c^	35.14 ± 0.64 ^ab^

**Table 6:** Antibacterial activity of aqueous flower extracts of N. nuda (prepared at 40 and 60 °C). Inhibition zone diameters (disk diffusion assay) and values for minimum inhibitory concentration (MIC) and minimum bactericidal concentration (MBC) are presented. Statistical differences between extracts obtained at 40 and 60 °C were evaluated using Student’s *t*-test (*n* > 3) and are indicated by asterisks (** *p* < 0.001).

	Inhibitory Zone (mm)		MIC (mg mL^−1^)		MBC (mg mL^−1^)		Positive Controls		
	Flower		Flower		Flower		Tetracycline (mm)	Gentamicin (µg mL^−1^)	Penicillin G (µg mL^−1^)
	40 °C	60 °C	40 °C	60 °C	40 °C	60 °C			
*Acinetobacter* *calcoaceticus*	11.5	10 **	25	25	50	25	16	1.25	-
*Bacillus* *subtilis*	14	13	6.25	12.5	6.25	12.5	50	-	<0.235
*Staphylococcus* *aureus*	9	4	12.5	6.25	25	12.5	32	-	<0.235

**Table 7:** Pearson correlation coefficients between metabolites identified in aqueous extracts of *N. nuda* flowers and biological activities, including antiviral activity against *Simplexvirus humanalpha2* (simultaneous application, SvHA2^SA^; 1 h post-treatment, SvHA2^PA^), anti-inflammatory activity (hemolysis and C1q assays), and antioxidant activity (DPPH). Only statistically significant correlations are shown (*p* < 0.01, *n* ≥ 3). For visualization, values are presented on a color scale ranging from white (minimum) to red (maximum).

Correlation Analysis	Anti- SvHA2^SA^	Anti- SvHA2^PA^	Anti- Inflammatory	C1q	Anti- Proliferative	DPPH
Phenols					0.994	0.995
Flavonoids					0.954	0.949
Chlorogenic acid					0.87	0.871
Caffeic acid					0.981	0.979
*p*-Coumaric acid	0.909	0.936	0.886	0.898		
Rosmarinic acid					0.994	0.994
Homovanillyl alcohol					0.939	0.939
Vanillic acid					0.973	0.978
Isoferulic acid					0.971	0.976
O-trans-p-Coumaroylglycerol					0.989	0.992
Isoorientin					0.997	0.997
Luteolin	0.983	0.987	0.996	0.992		
Apigenin	0.807	0.774	0.85	0.833		
Nepetanudoside	0.842	0.879	0.815	0.829		
Nepetalactone	0.998	0.997	0.993	0.998		
Propanoic acid	0.941	0.963	0.922	0.932		
Glycolic acid					0.996	0.997
Malonic acid					0.993	0.992
Citramalic acid					0.999	0.999
Alanine					0.929	0.935
Valine					0.983	0.985
Isoleucine					0.955	0.959
Glycine					0.908	0.902
Threonine					0.832	0.841
Cysteine	0.997	0.998	0.994	0.997		
Maltose					0.966	0.971
Cellobiose					0.92	0.927
Arabinose	0.899	0.873	0.929	0.918		
β-Gentiobiose	0.983	0.971	0.968	0.975		
Palmitic acid					0.984	0.983
Stearic acid					0.947	0.952

## Data Availability

Data are contained within the current article and its Supplementary Materials.

## Supplementary Materials

The following supporting information can be downloaded at: https://www.mdpi.com/article/10.3390/metabo16050323/s1, Table S1: Temperature-dependent yield and total content of phenolic compounds in aqueous extracts from *N. nuda* flower, leaf, stem; Table S2: Metabolites identified by GC–MS assay in aqueous extracts from *N. nuda* flower, leaf and stem; Table S3: Cytotoxicity of aqueous extracts from *N. nuda* flower, leaf, stem; Table S4: Biological activities of aqueous extracts from *N. nuda* flower, leaf, stem; Table S5: Data for cell viability (cytotoxicity) of the cell line used treated with and cell protection (antiviral activity) against SvHA2 (DD strain) of aqueous extracts of *N. nuda* flower, leaf, and stem, obtained at different temperatures; Table S6: Data for cell viability (cytotoxicity) of the cell line used and cell protection (antiviral activity) against SvHA2 (DD strain) by the positive control Acyclovir.

## Abbreviations

The following abbreviations are used in this manuscript:

ACV	acyclovir
astragalin	kaempferol-3-*O*-glucoside
C1q	complement system acting in the classical pathway of inflammation
CC_50_	50% cytotoxicity concentration
DW	dry weight
GC–MS	gas chromatography–mass spectrometry
IgG	immunoglobulin
isoorientin	luteolin-6-C-glycoside
HAIgG	heat-aggregated human IgG
MBC	minimum bactericidal concentration
MDBK	Madine and Darby bovine kidney (MDBK) cells
MIC	minimum inhibitory concentration
NBIMCC	National Bank for Industrial Microorganisms and Cell Cultures
PCA	principal component analysis
SI	selectivity index
SvHA2	*Simplexvirus humanalpha2*
SvHA2^SA^	SvHA2 simultaneous application
SvHA2^PA^	SvHA2 post-infection application
SWE	subcritical water extraction (SWE)
UHPLC/DAD/(±)HESI–MS^2^	ultra-high-performance liquid chromatography/diode-array detector/(±)heated electrospray ionization–tandem mass spectrometry
