# Supplementary material for: Extraction Temperatures Shape Water-Soluble Metabolite Profiles of Nepeta nuda L. and thus Modulate the Bioactive Properties
Source: Metabolites. 2026 May 13;16(5):323. doi: 10.3390/metabo16050323 (PMC13208640; doi:10.3390/metabo16050323)
Supplement: Supplementary file 1 [file metabolites-16-00323-s001.zip › metabolites-4263358-supplementary.pdf]

Supplementary Material

Table S1. Temperature-dependent yield and total content of phenolic compounds in aqueous extracts from *N. nuda* flower, leaf, stem. Data are presented as mean values and SE ($n \geq 3$). Statistical differences between the variants were determined by two-way ANOVA followed by Tukey's post hoc test and are indicated with different letters. For visualization, values are represented on a color scale ranging from white (minimum) to red (maximum).

Extraction t °C	30 °C	40 °C	50 °C	60 °C
**Yield %**				
Flower	16.6±0.5 [b]	16.6±1.8 [b]	17.1±1.2 [bc]	20.7±0.7 [d]
Leaf	16.8±0.8 [bc]	19.7±0.4 [d]	19.3±0.8 [cd]	21.4±0.6 [d]
Stem	10.9±0.6 [a]	11.9±0.5 [a]	12.3±0.4 [a]	12.9±0.5 [a]
**Phenols mg g DW^{-1}**				
Flower	61.26±0.67 [bc]	70.24±0.65 [d]	80.98±0.09 [e]	91.07±0.56 [g]
Leaf	50.89±0.42 [a]	63.67±1.40 [c]	87.09±0.40 [f]	104.69±0.88 [h]
Stem	58.39±0.58 [b]	60.06±1.21 [b]	61.81±0.24 [bc]	68.30±0.81 [d]
**Flavonoids mg g DW^{-1}**				
Flower	34.74±0.92 [a]	40.26±0.40 [b]	41.75±0.70 [bc]	43.51±0.35 [bcd]
Leaf	44.74±0.40 [cd]	55.26±0.26 [e]	76.93±0.99 [f]	84.82±1.52 [g]
Stem	42.63±0.85 [bc]	43.16±0.70 [bcd]	44.74±0.15 [cd]	46.67±0.53 [d]

Table S2. Metabolites identified by GC–MS assay in aqueous extracts from *N. nuda* flower, leaf and stem. (**a**) Mass spectral data. (**b**) Relative values to the control (Flower 40 °C) are represented under logarithm. Statistical differences between the variants for each metabolite were determined using a two-way ANOVA followed by Tukey's post hoc test and are indicated with different letters. For visualization, values for each metabolite are presented on a color scale ranging from white (minimum) to red (maximum).

(a)

		RT	RI	Mass spectral data
	**Organic acids**			
P1	Propanoic acid	4.846	1048.20	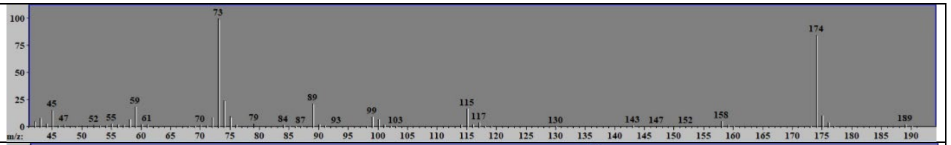
P2	Glycolic acid	5.1120	1073.6	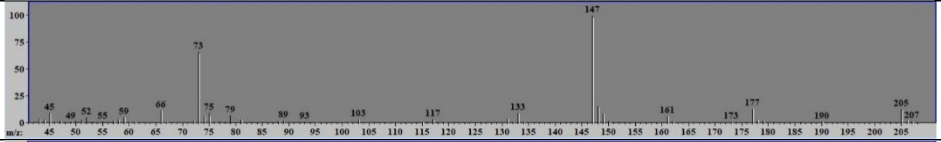
P3	Malonic acid	6.511	1198.2	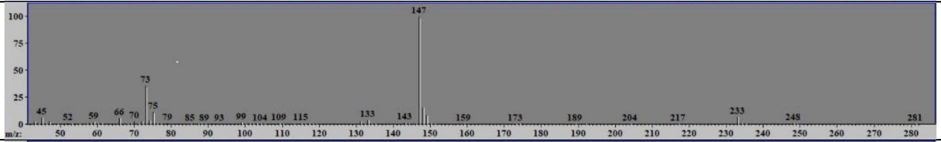
P4	Succinic acid	7.611	1311.2	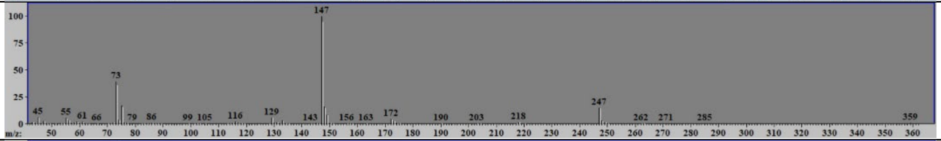
P5	Glyceric acid	7.733	1323.7	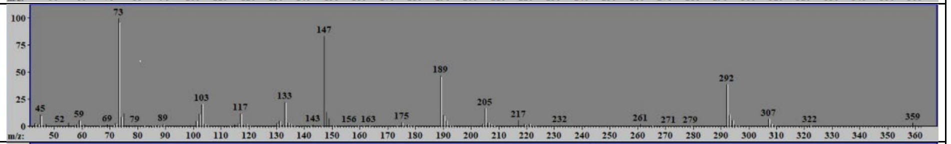
P6	Fumaric acid	7.952	1346.3	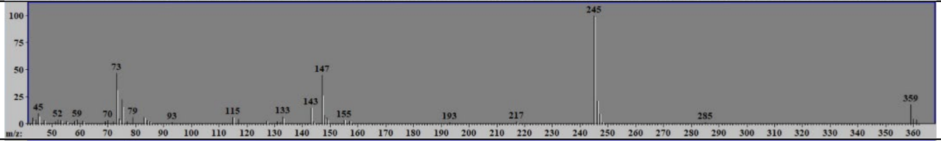
P7	Butanoic acid	8.667	1418.9	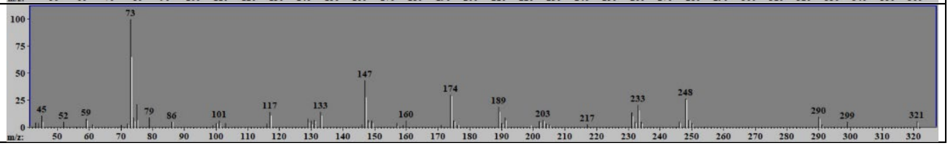

		RT	RI	Mass spectral data
P8	Citramalic acid	9.050	1456.5	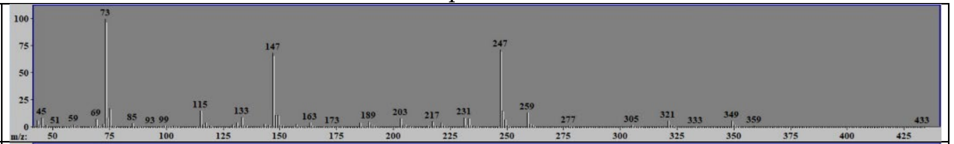
P9	Malic acid	9.191	1470.5	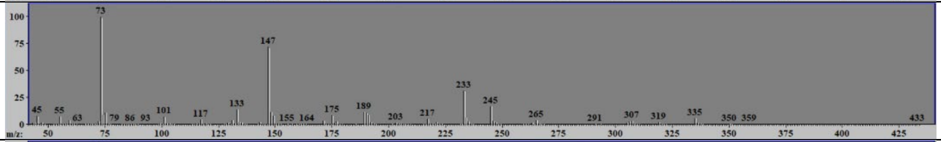
P10	Erythronic acid	9.897	1539.8	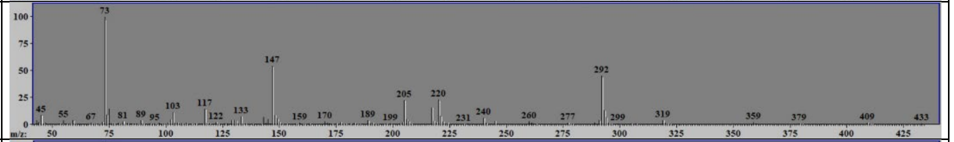
P11	Tartaric acid	10.794	1621.4	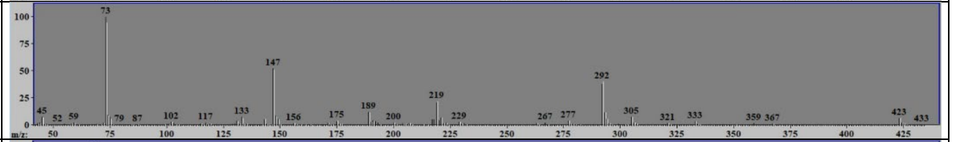
P12	Citric acid	13.298	1808.9	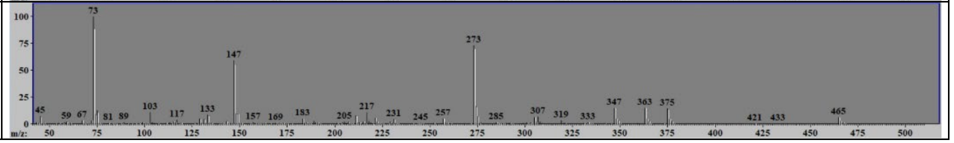
	**Amino acids**			
P13	Alanine	5.442	1101.9	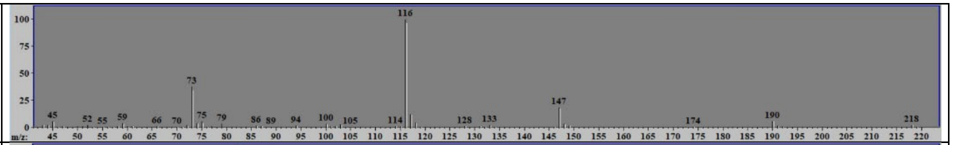
P14	Leucine	6.049	1156.6	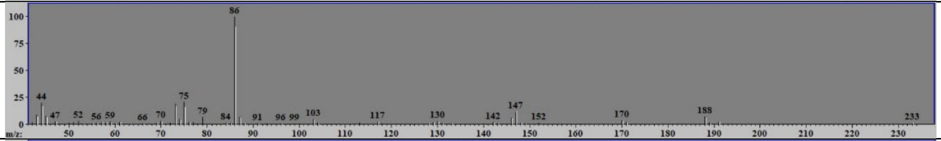
P15	Valine	6.639	1211.1	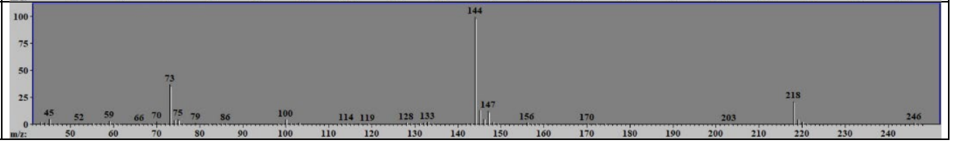

		RT	RI	Mass spectral data
P16	Isoleucine	7.410	1290.4	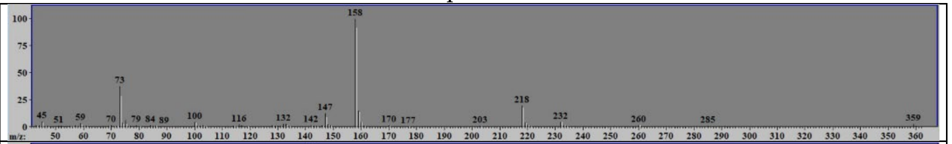
P17	Proline	7.481	1297.7	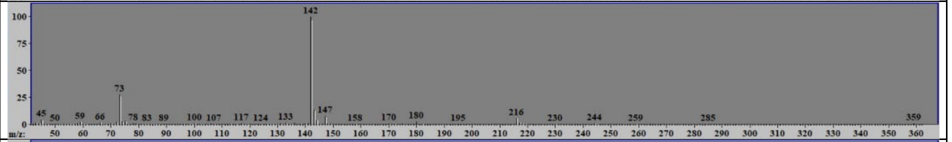
P18	Glycine	7.551	1305.0	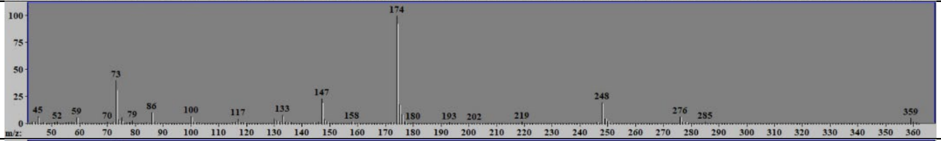
P19	Serine	8.024	1353.6	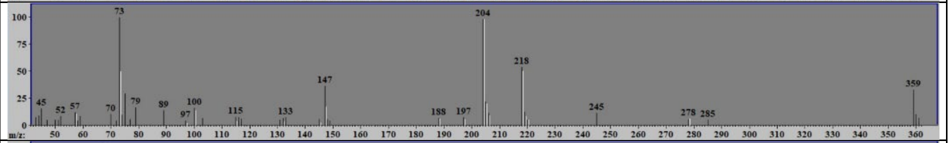
P20	Threonine	8.255	1377.5	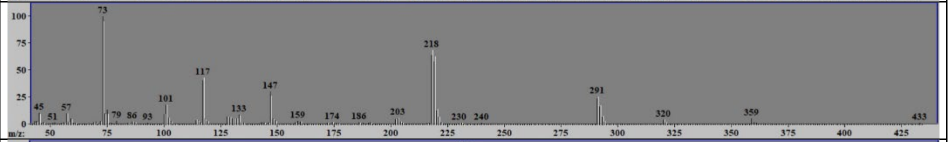
P21	Aspartic acid	9.514	1502.2	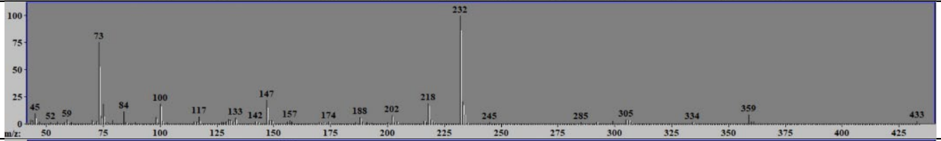
P22	5-Oxoproline	9.589	1509.5	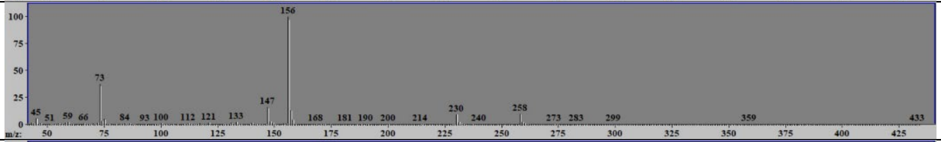
P23	γ-Aminobutyric acid (GABA)	9.662	1516.7	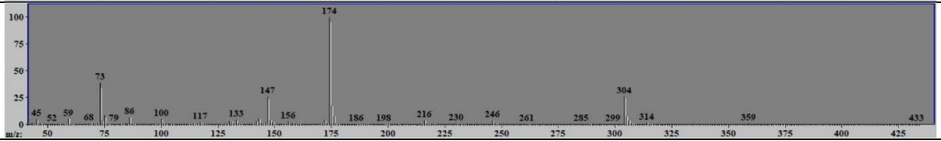

		RT	RI	Mass spectral data
P24	Cysteine	11.762	1694.5	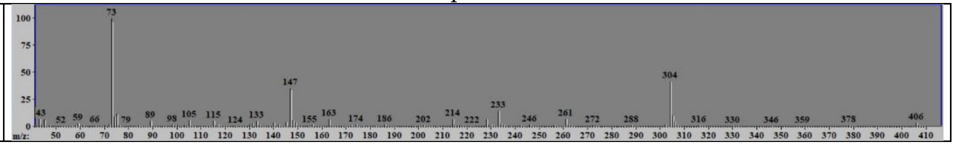
	**Sugar derivatives**			
P25	Glycerol	7.194	1268.3	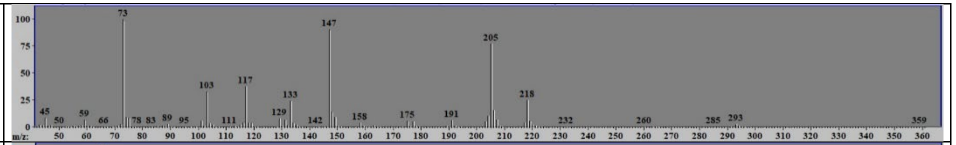
P26	Arabinose	11.107	1645.0	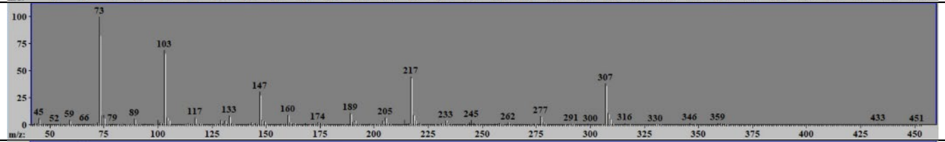
P27	Fructose	14.140	1863.1	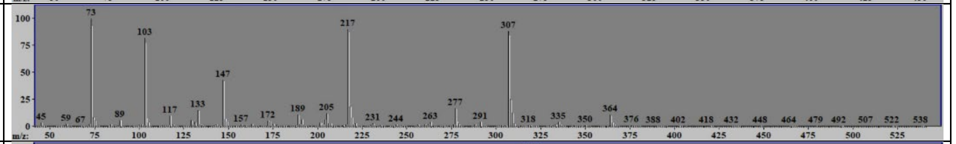
P28	Glucose	14.559	1890.1	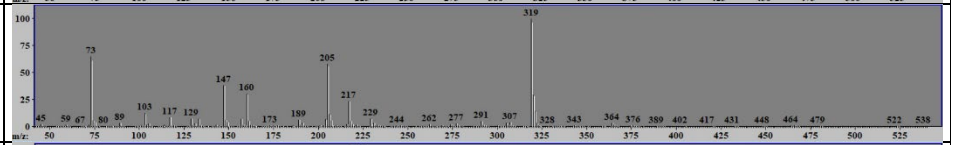
P29	Myo-Inositol	17.621	2083.2	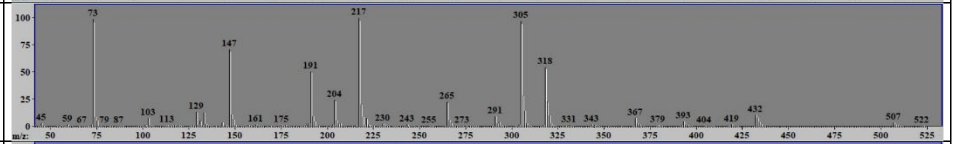
P30	Maltose	24.256	2500.7	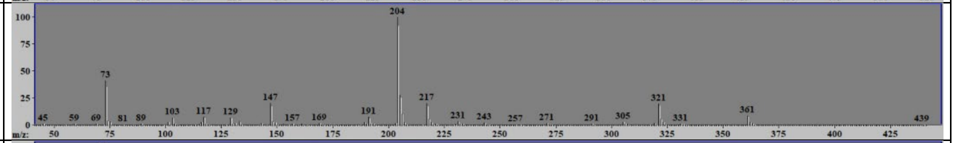
P31	Sucrose	26.185	2629.2	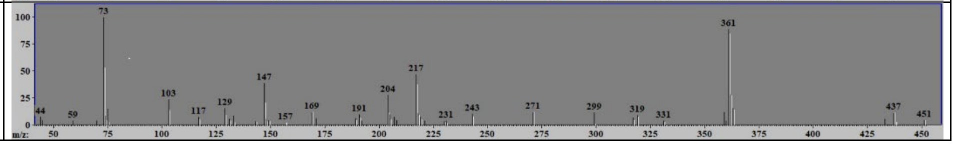

		RT	RI	Mass spectral data
P32	Cellobiose	27.619	2729.4	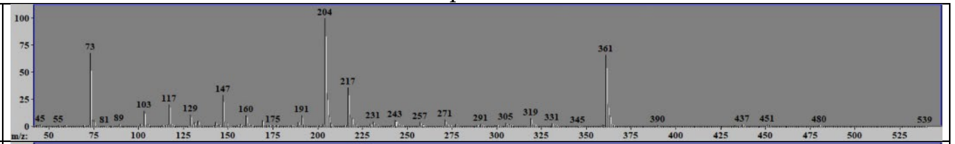
P34	β-Gentiobiose	28.882	2818.8	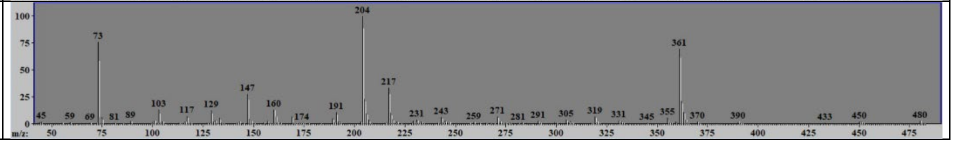
	**Phenolic derivatives**			
P35	Homovanillyl alcohol	11.794	1 696.9	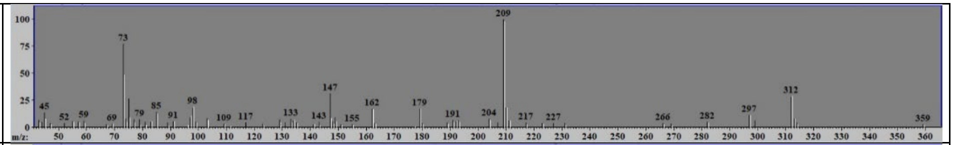
P36	Vanillic acid	12.564	1 755.0	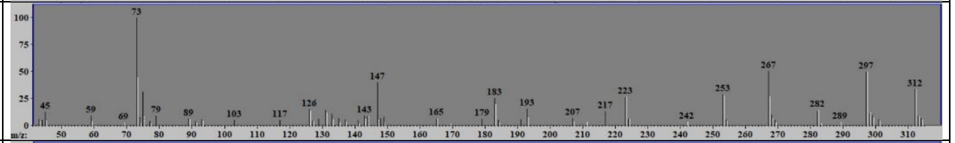
P37	Isoferulic acid	17.675	2 086.4	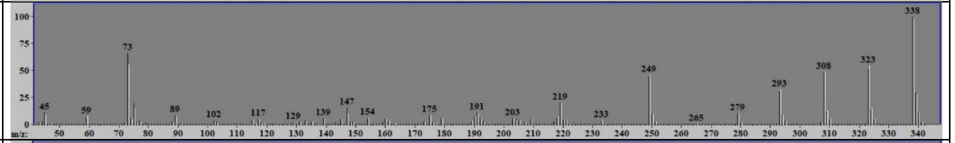
P38	Caffeic acid	18.419	2132.0	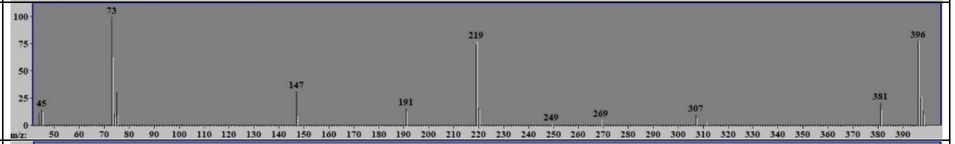
P39	O-trans-p-Coumaroylglycerol	24.033	2486.1	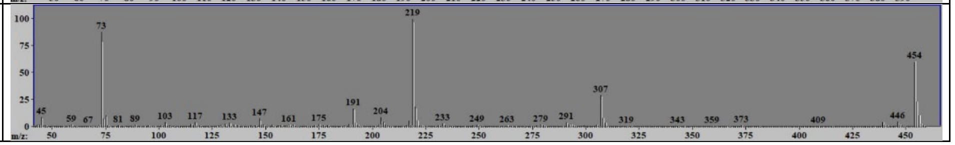
	**Others**			
P40	Quininic acid	13.884	1846.7	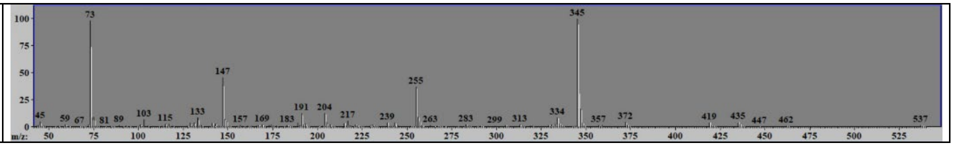

		RT	RI	Mass spectral data
	Fatty acids and derivatives			
NP1	Palmitic acid	15.0343	1922.5	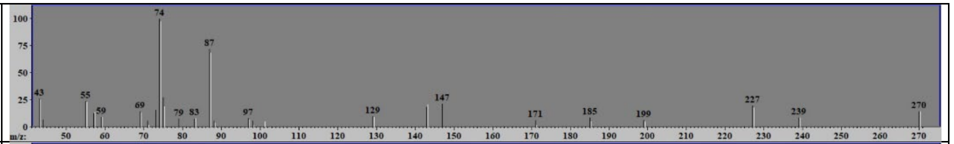
NP2	Methyl stearate	18.2742	2125.1	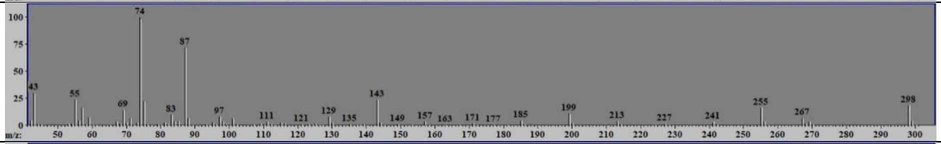
NP3	Stearic acid	20.1447	2240.7	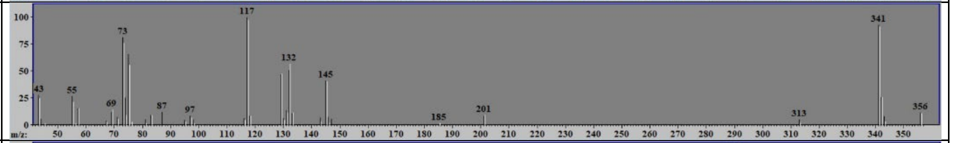
NP4	Monopalmitin	25.5234	2584.5	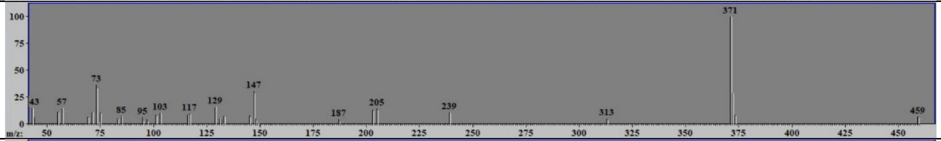
NP5	Monostearin	27.827	2743.90	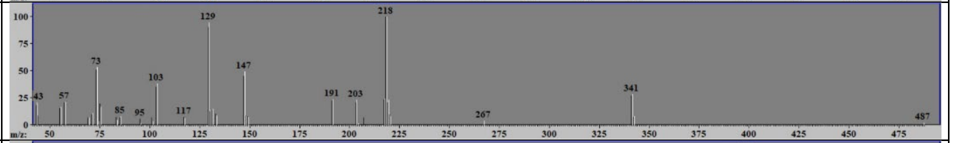
NP6	Glycerol monostearate	28.2848	2778.2	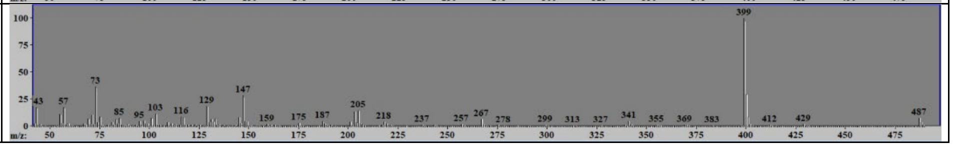

(b)

		RT	RI	Flower		Leaf		Stem	
				40 °C	60 °C	40 °C	60 °C	40°C	60 °C
	**Organic acids**								
P1	Propanoic acid	4.846	1048.20	0^{ab}	-0.27^{a}	2.06^{c}	2.73^{d}	-0.02^{ab}	0.21^{b}
P2	Glycolic acid	5.1120	1073.6	0^{b}	0.38^{d}	-0.27^{a}	0.26^{cd}	0.11^{bc}	1.04^{e}
P3	Malonic acid	6.511	1198.2	0^{d}	0.72^{e}	-0.34^{c}	-0.01^{d}	-1.33^{a}	-0.62^{b}
P4	Succinic acid	7.611	1311.2	0^{a}	0.01^{a}	0.52^{b}	0.46^{b}	0.72^{b}	1.05^{c}
P5	Glyceric acid	7.733	1323.7	0^{a}	0.00^{a}	0.19^{ab}	0.12^{ab}	0.27^{b}	0.68^{c}
P6	Fumaric acid	7.952	1346.3	0^{ab}	0.21^{b}	-0.19^{a}	0.32^{b}	0.70^{c}	1.31^{d}
P7	Butanoic acid	8.667	1418.9	0^{bc}	0.29^{c}	-0.44^{a}	-0.21^{ab}	-0.46^{a}	-0.06^{bc}
P8	Citramalic acid	9.050	1456.5	0^{a}	0.73^{c}	0.29^{b}	0.35^{b}	0.21^{ab}	0.62^{c}
P9	Malic acid	9.191	1470.5	0^{c}	-0.05^{c}	-0.65^{b}	-1.04^{a}	0.26^{d}	0.70^{e}
P10	Erythronic acid	9.897	1539.8	0^{a}	0.02^{a}	0.44^{b}	0.56^{b}	0.80^{c}	1.25^{d}
P11	Tartaric acid	10.794	1621.4	0^{b}	0.22^{bc}	-0.70^{a}	-0.56^{a}	0.40^{c}	0.79^{d}
P12	Citric acid	13.298	1808.9	0^{d}	-0.12^{d}	-1.55^{a}	-1.60^{a}	-0.88^{b}	-0.41^{c}
	**Amino acids**								
P13	Alanine	5.442	1101.9	0^{ab}	1.27^{d}	0.33^{b}	1.04^{cd}	-0.43^{a}	0.40^{bc}
P14	Leucine	6.049	1156.6	0^{b}	-0.11^{b}	-0.54^{b}	-0.40^{b}	-1.33^{a}	-1.61^{a}
P15	Valine	6.639	1211.1	0^{b}	0.88^{c}	0.14^{b}	0.60^{c}	-0.76^{a}	-0.02^{b}
P16	Isoleucine	7.410	1290.4	0^{b}	0.80^{d}	0.09^{bc}	0.49^{cd}	-0.69^{a}	-0.20^{b}
P17	Proline	7.481	1297.7	0^{ab}	1.36^{c}	-0.83^{a}	-0.02^{ab}	0.39^{bc}	1.23^{c}
P18	Glycine	7.551	1305.0	0^{c}	0.45^{d}	-0.38^{b}	-0.37^{b}	-1.68^{a}	-1.37^{a}
P19	Serine	8.024	1353.6	-1^{ab}	1.31^{b}	-1.49^{a}	-0.45^{ab}	-1.52^{a}	-0.71^{ab}
P20	Threonine	8.255	1377.5	0^{ab}	0.91^{c}	0.17^{bc}	0.55^{bc}	-0.74^{a}	-0.06^{ab}
P21	Aspartic acid	9.514	1502.2	0^{bc}	0.29^{c}	-1.52^{a}	-1.39^{a}	-1.29^{a}	-0.61^{b}
P22	5-Oxoproline	9.589	1509.5	0^{c}	0.36^{c}	-1.67^{a}	-1.16^{b}	-1.89^{a}	-0.99^{b}
P23	γ-Aminobutyric acid (GABA)	9.662	1516.7	0^{bc}	0.06^{bc}	0.32^{d}	0.13^{cd}	-0.51^{a}	-0.14^{b}
P24	Cysteine	11.762	1694.5	0^{b}	-1.26^{a}	1.22^{e}	0.22^{bc}	0.47^{d}	0.42^{cd}
	**Sugar derivatives**								
P25	Glycerol	7.194	1268.3	0^{c}	0.00^{c}	-0.33^{b}	-0.35^{b}	-0.55^{a}	-0.03^{c}
P26	Arabinose	11.107	1645.0	0^{c}	-0.53^{a}	-0.10^{c}	-0.50^{ab}	-0.22^{bc}	-0.58^{a}
P27	Fructose	14.140	1863.1	0^{b}	0.00^{b}	-0.32^{a}	-0.32^{a}	-0.29^{a}	0.02^{b}
P28	Glucose	14.559	1890.1	0^{bc}	-0.02^{bc}	-0.09^{abc}	-0.16^{ab}	-0.25^{a}	0.05^{c}
P29	Myo-Inositol	17.621	2083.2	0^{c}	-0.01^{c}	0.03^{c}	-0.01^{c}	-0.82^{a}	-0.54^{b}
P30	Maltose	24.256	2500.7	0^{c}	0.15^{c}	0.99^{d}	0.98^{d}	-1.14^{a}	-0.71^{b}
P31	Sucrose	26.185	2629.2	0^{a}	0.18^{ab}	0.38^{b}	0.51^{b}	0.14^{ab}	1.52^{c}
P32	Cellobiose	27.619	2729.4	0^{cd}	0.21^{d}	-0.24^{c}	-0.11^{c}	-2.07^{a}	-0.65^{b}
P34	β-Gentiobiose	28.882	2818.8	0^{bc}	-0.58^{a}	0.45^{d}	0.16^{cd}	-0.07^{bc}	-0.22^{b}
	**Phenolic derivatives**								
P35	Homovanillyl alcohol	11.794	1 696.9	0^{b}	0.47^{b}	-0.93^{a}	-0.03^{b}	n.a.	n.a.
P36	Vanillic acid	12.564	1 755.0	0^{ab}	1.31^{c}	0.28^{b}	0.41^{b}	-0.64^{a}	0.68^{bc}
P37	Isoferulic acid	17.675	2 086.4	0^{c}	0.52^{d}	0.19^{cd}	0.50^{d}	-1.05^{a}	-0.45^{b}
P38	Caffeic acid	18.419	2132.0	0^{c}	2.42^{e}	-1.44^{b}	1.36^{d}	-3.19^{a}	0.04^{c}
P39	O-trans-p-Coumaroylglycerol	24.033	2486.1	0^{c}	0.92^{d}	0.15^{c}	1.65^{e}	-2.42^{a}	-1.05^{b}
	**Others**								
P40	Quininic acid	13.884	1846.7	0^{bc}	0.01^{bc}	-1.07^{a}	-0.80^{a}	-0.07^{b}	0.37^{c}
	**Fatty acids and derivatives**								
NP1	Palmitic acid	15.0343	1922.5	0^{a}	1.24^{b}	0.37^{a}	0.31^{a}	2.24^{c}	2.11^{c}
NP2	Methyl stearate	18.2742	2125.1	0^{a}	0.50^{a}	0.08^{a}	0.15^{a}	0.58^{a}	0.65^{a}
NP3	Stearic acid	20.1447	2240.7	0^{a}	1.27^{b}	0.35^{ab}	0.97^{b}	0.83^{ab}	1.01^{b}
NP4	Monopalmitin	25.5234	2584.5	0^{ab}	-0.18^{ab}	0.16^{ab}	0.50^{b}	0.08^{ab}	-0.45^{a}
NP5	Monostearin	27.827	2743.90	0^{abc}	-1.33^{a}	-0.46^{ab}	0.27^{abc}	1.34^{c}	0.95^{bc}
NP6	Glycerol monostearate	28.2848	2778.2	0^{a}	-0.27^{a}	0.11^{a}	0.53^{a}	0.36^{a}	0.17^{a}

Table S3. Cytotoxicity of aqueous extracts from *N. nuda* flower, leaf, stem. Bovine kidney cells (MDBK) were exposed to treatments with series of extracts concentrations. Data are presented as mean values and SE ($n \geq 3$). Statistical differences between the variants were determined by two-way ANOVA followed by Tukey's post hoc test and are indicated with different letters. For visualization, values are represented on a color scale ranging from white (minimum) to red (maximum). CC_{50} – extract concentration required for the reduction of cell viability by 50%.

**Extraction t °C**	**30 °C**	**40 °C**	**50 °C**	**60 °C**
**Cytotoxicity (mg mL^{-1}), CC_{50}**				
Flower	6.20±0.49 ^{abc}	5.57±0.57 ^{ab}	5.40±0.43 ^{a}	6.24±0.55 ^{abcd}
Leaf	6.50±0.68 ^{abcd}	6.85±0.74 ^{bcd}	6.44±0.33 ^{abcd}	7.64±0.25 ^{d}
Stem	5.75±0.21 ^{ab}	6.22±0.48 ^{abcd}	7.26±0.34 ^{cd}	7.40±0.49 ^{cd}

Table S4. Biological activities of aqueous extracts from *N. nuda* flower, leaf, stem. Data are presented as mean values and SE ($n \geq 3$). Statistical differences between the variants were determined by two-way ANOVA followed by Tukey's post hoc test and are indicated with different letters. For visualization, values are represented on a color scale ranging from white (minimum) to red (maximum).

**Extraction t °C**	**30 °C**	**40 °C**	**50 °C**	**60 °C**
**DPPH mM g DW^{-1}**				
Flower	448.80± 4.27 ^{b}	652.51± 5.70 ^{c}	973.02± 2.14 ^{d}	1172.45± 4.27 ^{e}
Leaf	205.21± 2.85 ^{a}	391.82± 0.71 ^{b}	950.23±12.82 ^{d}	1269.79±92.97 ^{e}
Stem	316.32±11.40 ^{ab}	437.41± 2.14 ^{b}	596.95± 9.97 ^{c}	668.18±12.82 ^{c}
**Anti-inflammatory activity % (AI)**				
Flower	70.58±1.30 ^{bc}	90.72±4.40 ^{cd}	20.22±7.50 ^{a}	18.30±10.60 ^{a}
Leaf	52.24±2.30 ^{b}	98.83±5.40 ^{d}	22.91±8.50 ^{a}	26.30±11.60 ^{a}
Stem	87.42±3.30 ^{cd}	90.29±6.40 ^{cd}	53.53±9.50 ^{b}	28.54±12.60 ^{a}
**Antiviral protection % simultaneous application (SvHA2^{SA})**				
Flower	93.90±12.8 ^{d}	89.93±12.9 ^{cd}	69.43±2.61 ^{ab}	65.68±3.98 ^{a}
Leaf	85.45±2.89 ^{bcd}	84.59±0.33 ^{bcd}	76.38±4.29 ^{abcd}	68.64±2.79 ^{ab}
Stem	90.13±8.40 ^{cd}	88.38±2.58 ^{cd}	77.82±4.11 ^{abcd}	72.50±0.70 ^{abc}
**Antiviral protection % post-infection application (SvHA2^{PA})**				
Flower	59.68±0.27 ^{de}	68.29±6.51 ^{e}	47.19±0.56 ^{bcd}	28.22±2.33 ^{a}
Leaf	50.28±2.69 ^{cd}	37.18±7.76 ^{abc}	30.20±1.55 ^{a}	41.25±1.06 ^{abc}
Stem	60.61±12.5 ^{de}	56.61±4.86 ^{de}	32.99±2.39 ^{a}	35.14±0.64 ^{ab}

Table S5. Data for cell viability (cytotoxicity) of the cell line used treated with and cell protection (antiviral activity) against SvHA2 (DD strain) of aqueous extracts of *N. nuda* flower, leaf and stem, obtained at different temperatures.

	Cell viability (Cytotoxicity)						Cell protection (Antiviral activity)																	
							Test sample added simultaneously with inoculation of cell monolayer									Test sample added 1 hour after inoculation of cell monolayer								
	Flower		Leaf		Stem		Flower			Leaf			Stem			Flower			Leaf			Stem		
	MNC[a,b] mg mL^{-1}	CC$_{50}$[a,b] mg mL^{-1}	MNC[a,b] mg mL^{-1}	CC$_{50}$[a,b] mg mL^{-1}	MNC[a,b] mg mL^{-1}	CC$_{50}$[a,b] mg mL^{-1}	Protection % in MNC[a,b]	EC$_{50}$[a,b] mg mL^{-1}	**SI**	Protection % in MNC[a,b]	EC$_{50}$[a,b] mg mL^{-1}	**SI**	Protection % in MNC[a,b]	EC$_{50}$[a,b] mg mL^{-1}	**SI**	Protection % in MNC[a,b]	EC$_{50}$[a,b] mg mL^{-1}	**SI**	Protection % in MNC[a,b]	EC$_{50}$[a,b] mg mL^{-1}	**SI**	Protection % in MNC[a,b]	EC$_{50}$[a,b] mg mL^{-1}	**SI**
30°C	1	6.20 (±0.49)	0.75	6.50 (±0.68)	0.75	5.75 (±0.21)	93.9 (±12.8)	0.37 (±0.13)	**16.75**	85.45 (±2.89)	0.37 (±0.69)	**17.56**	90.13 (±8.40)	0.33 (±0.15)	**17.42**	59.68 (±0.27)	0.80 (±0.41)	**7.75**	50.28 (±2.69)	0.74 (±0.16)	**8.78**	60.61 (±12.5)	0.57 (±0.24)	**10.08**
40°C	1	5.57 (±0.57)	0.75	6.85 (±0.74)	0.75	6.22 (±0.48)	89.93 (±12.9)	0.39 (±0.16)	**14.28**	84.59 (±0.33)	0.40 (±0.67)	**17.12**	88.38 (±2.58)	0.40 (±0.20)	**15.55**	68.29 (±6.51)	0.78 (±0.97)	**7.14**	37.18 (±7.76)	n.d.	**n.d.**	56.61 (±4.86)	0.66 (±0.17)	**9.42**
50°C	2	5.40 (±0.43)	1	6.44 (±0.33)	1	7.26 (±0.34)	69.43 (±2.61)	0.89 (±0.32)	**6.06**	76.38 (±4.29)	0.67 (±0.39)	**9.61**	77.82 (±4.11)	0.43 (±0.12)	**16.88**	47.19 (±0.56)	n.d.	**n.d.**	30.2 (±1.55)	n.d.	**n.d.**	32.99 (±2.39)	n.d.	**n.d.**
60°C	2	6.24 (±0.55)	1.75	7.64 (±0.25)	1.5	7.40 (±0.49)	65.68 (±3.98)	1.51 (±0.58)	**4.13**	68.64 (±2.79)	0.94 (±0.52)	**8.12**	72.50 (±0.70)	0.68 (±0.79)	**10.88**	28.22 (±2.33)	n.d.	**n.d.**	41.25 (±1.06)	n.d.	**n.d.**	35.14 (±0.64)[5]	n.d.	**n.d.**

MNC—maximum nontoxic concentration; CC$_{50}$—cytotoxic concentration$_{50}$; EC$_{50}$—effective concentration$_{50}$; SI – selective index; n.d. – not detected;
[a] The results are expressed as the mean value; [b] The parentheses represented standard deviation (±SD) (different from 0).

Table S6. Data for cell viability (cytotoxicity) of the cell line used and cell protection (antiviral activity) against SvHA2 (DD strain) by the positive control Acyclovir.

Cell viability (Cytotoxicity)		Cell protection (Antiviral activity)					
		Test sample added simultaneously with inoculation of cell monolayer			Test sample added 1 hour after inoculation of cell monolayer		
MNC[a,b] mg mL^{-1}	CC$_{50}$[a,b] mg mL^{-1}	Prot. (%) in MNC[a,b]	EC$_{50}$[a,b] mg mL^{-1}	SI	Prot. (%) in MNC[a,b]	EC$_{50}$[a,b] mg mL^{-1}	SI
0.0312	0.341 (±0.02)	15.395 (±2.19)	n.d.	n.d.	9.2945 (±2.6)	n.d.	n.d.

MNC—maximum nontoxic concentration; CC$_{50}$—cytotoxic concentration$_{50}$; EC$_{50}$—effective concentration$_{50}$; SI – selective index; n.d. – not detected;
[a] The results are expressed as the mean value; [b] The parentheses represented standard deviation (±SD) (different from 0).
